# Investigating imaging network markers of cognitive dysfunction and pharmacoresistance in newly diagnosed epilepsy: a protocol for an observational cohort study in the UK

**DOI:** 10.1136/bmjopen-2019-034347

**Published:** 2019-10-16

**Authors:** Christophe de Bézenac, Marta Garcia-Finana, Gus Baker, Perry Moore, Nicola Leek, Rajiv Mohanraj, Leonardo Bonilha, Mark Richardson, Anthony Guy Marson, Simon Keller

**Affiliations:** 1 Department of Molecular and Clinical Pharmacology, Institute of Translational Medicine, University of Liverpool, Liverpool, UK; 2 Department of Biostatistics, University of Liverpool, Liverpool, UK; 3 Department of Neurology, The Walton Centre NHS Foundation Trust, Liverpool, UK; 4 Department of Neurology, Salford Royal NHS Foundation Trust, Salford, UK; 5 Department of Neurology, Medical University of South Carolina, Charleston, South Carolina, USA; 6 Institute of Psychiatry, Psychology and Neuroscience, King's College London, London, UK

**Keywords:** newly diagnosed, epilepsy, brain connectivity, neuropsychology, inflammation

## Abstract

**Introduction:**

Epilepsy is one of the most common serious brain disorders, characterised by seizures that severely affect a person’s quality of life and, frequently, their cognitive and mental health. Although most existing work has examined chronic epilepsy, newly diagnosed patients present a unique opportunity to understand the underlying biology of epilepsy and predict effective treatment pathways. The objective of this prospective cohort study is to examine whether cognitive dysfunction is associated with measurable brain architectural and connectivity impairments at diagnosis and whether the outcome of antiepileptic drug treatment can be predicted using these measures.

**Methods and analysis:**

107 patients with newly diagnosed focal epilepsy from two National Health Service Trusts and 48 healthy controls (aged 16–65 years) will be recruited over a period of 30 months. Baseline assessments will include neuropsychological evaluation, structural and functional Magnetic Resonance Imaging (MRI), Electroencephalography (EEG), and a blood and saliva sample. Patients will be followed up every 6 months for a 24-month period to assess treatment outcomes. Connectivity- and network-based analyses of EEG and MRI data will be carried out and examined in relation to neuropsychological evaluation and patient treatment outcomes. Patient outcomes will also be investigated with respect to analysis of molecular isoforms of high mobility group box-1 from blood and saliva samples.

**Ethics and dissemination:**

This study was approved by the North West, Liverpool East Research Ethics Committee (19/NW/0384) through the Integrated Research Application System (Project ID 260623). Health Research Authority (HRA) approval was provided on 22 August 2019. The project is sponsored by the UoL (UoL001449) and funded by a UK Medical Research Council (MRC) research grant (MR/S00355X/1). Findings will be presented at national and international meetings and conferences and published in peer-reviewed journals.

**Trial registration number:**

IRAS Project ID 260623.

**Strengths and limitations of this study**

- This will be the first study to prospectively investigate brain structural and physiological architecture and connectivity in adults with a new diagnosis of focal epilepsy.
- The study is expected to provide insights into the biology underlying cognitive dysfunction in the early stages of human epilepsy, and to lead to the development of prognostic markers of future pharmacoresistance.
- Expected recruitment has been based on records of past diagnosis at recruitment sites and while the study is expected to recruit well, unexpected under-recruitment is possible and would be a barrier to timely completion.
- A second potential limitation of this study is the potential for participant attrition and loss of patient follow-up at multiple points over 24 months; missing data could impact on the validity of study conclusions.

## Introduction

### Background and rationale

Epilepsy is one of the most common serious brain disorders; every day in the UK, 87 people are diagnosed with epilepsy, affecting over 600 000 people.^1^ The condition is characterised by devastating seizures that severely impact on a person’s quality of life. Epilepsy frequently affects a person’s cognitive and mental health,^2^ and the disorder contributes to elevated propensity for depression, suicide and sudden and unexpected death compared with the general population.^3 4^ Despite this, research into epilepsy has been grossly underfunded compared with other medical conditions of similar economic, social and personal impact.^5^ The vast majority of existing work in human studies has been performed in chronic epilepsy. Newly diagnosed epilepsy (NDE) is only rarely studied despite representing a key point in time to understand the underlying biology of the disorder in the absence of confounds including antiepileptic drugs (AEDs) and long-term seizure effects.^6^ It is important to understand the reasons why people with epilepsy experience cognitive problems and seizures after treatment using safe imaging technologies from the earliest time point of the disorder. If we can understand these reasons in the early stages of epilepsy, we may be able to predict which patients will continue to experience seizures despite standard drug therapy. Patients who will not respond to drug therapy could potentially be offered alternative or adjunctive treatments, saving time, cost and the experience of undesirable side effects of certain AEDs.

Magnetic Resonance Imaging (MRI) and Electroencephalography (EEG) are routinely used to assess people with epilepsy. However, the application of these brain imaging techniques in the context of standard care cannot determine why some patients have cognitive problems and why others do not, and why some patients do not respond to AED therapy while others do. A new direction of brain imaging is therefore required, preferably one that can be incorporated into the standard clinical evaluation of patients. In patients with longstanding epilepsy the study of brain connectivity and networks (how different regions of the brain work together by virtue of their connectivity) using MRI and EEG has recently provided valuable insights into how the brain is structurally and physiologically altered in epilepsy.^7 8^ There is growing evidence that aberrant network dynamics are a key part of the underlying mechanisms of focal and generalised epilepsies.^9^ State-of-the-art quantitative structural (eg, diffusion MRI and tractography approaches), functional (eg, resting-state functional MRI (fMRI)), and physiological (eg, EEG) imaging techniques have provided a novel way of automatically distinguishing longstanding epilepsy patients from healthy controls,^10^ and predicting postoperative treatment outcome in severe epilepsy.^11–16^ We propose that these approaches will provide new explanations for the causes of cognitive problems and future treatment outcome from the beginning of a patient’s life with epilepsy.

Furthermore, mechanistic blood and saliva biomarkers could greatly enhance drug discovery by providing novel therapeutic targets and enrich trial populations, facilitating early surgical evaluation in drug resistance. We have soon-to-be-published data suggesting that molecular isoforms of high mobility group Box 1 (HMGB1) – a protein critically involved in the initiation of the inflammatory cascade in epilepsy –17 have potential as a prognostic biomarker. The acetylated, disulfide form of HMGB1, which triggers pro-inflammatory cytokine release via toll-like receptor 4, has shown pathological effects in pre-clinical models of seizures.^18^ In a parallel running study, we are currently studying people with longstanding epilepsy using MRI and blood serum markers of HMGB1 (short title: ‘MRI of inflammation in epilepsy’; IRAS project ID 220138; REC reference 17/NW/0342, Northwest-Liverpool).

This observational cohort study will be the first to prospectively investigate brain structural and physiological architecture and connectivity in adults with NDE with overarching goals to: (1) understand the neural basis of cognitive impairment; and (2) identify why and in whom seizures persist despite AED therapy. We will recruit adults with a new diagnosis of focal epilepsy and perform cognitive assessment and sophisticated analysis of MRI and EEG data. At the time of scanning and neuropsychological evaluation (baseline), all participants will additionally have blood and saliva extracted. Patients will be followed up longitudinally to determine their response to AED therapy. MRI/EEG data will be used to identify the neural correlates of cognitive impairment and to predict treatment outcome. Data generated from extracted blood and saliva samples will also be used to predict treatment outcome. To remain consistent with our ongoing work that investigates the correlation between MRI data in HMGB1 in people with epilepsy, we will use an identical approach of data acquisition and analysis. This work will be performed in an environment with demonstrated excellence in the care of people with epilepsy, recruitment of adults with NDE into clinical trials and expertise in MRI, EEG, neuropsychological and blood serum analysis. The research objectives of the proposed work directly address internationally agreed research priorities in epilepsy, with potential to provide significant insights into the epilepsy phenotype and to generate clinically meaningful non-invasive markers of treatment outcome.^19 20^

### Study objectives and design

The goal of the proposed research is to perform the first prospective multi-modal imaging investigation of brain architecture and connectivity in adults with a new diagnosis of focal epilepsy. The project aims to provide new insights into the biology underlying cognitive dysfunction in the early stages of human epilepsy and develop prognostic markers of future pharmacoresistance. The research will take place in context of a collaborative research and clinical environment that has demonstrated excellence in the recruitment and study of patients with NDE. The three main objectives are outlined below.

#### Objective 1

The primary objective is to determine the cognitive phenotype associated with NDE and whether cognitive dysfunction is associated with measurable brain architectural and connectivity impairments at diagnosis. We expect that patients will be cognitively impaired in the domains of memory, sustained attention and executive function; this impairment will be reflected in pathological alterations to structural and functional neural networks and responses to a verbal memory task computed from f/MRI and EEG.

#### Objective 2

A secondary objective is to determine whether AED treatment outcome can be predicted using multi-modal imaging measures of brain architecture and connectivity at the point of epilepsy diagnosis. We expect that architectural and physiological alterations within local and networked brain regions can predict patient response to pharmacological therapy at diagnosis.

#### Objective 3

We will determine whether blood serum and saliva derived measures of inflammation can predict AED treatment outcome in patients with NDE and examine relationships between molecular isoforms of HMGB1 and MRI, EEG and neuropsychological data.

## Methods and analysis

### Study environment

Research will be carried out by the Epilepsy Research Group within the Institute of Translational Medicine, University of Liverpool (UoL). The group is closely affiliated with the Walton Centre Foundation NHS Trust (WCFT) from where patients will be recruited, alongside the Salford Royal NHS Foundation Trust (SRFT). Both WCFT and SRFT will acquire patient EEG data in context of standard clinical care; EEG data for healthy controls will be acquired at the WCFT. MRI acquisition will be performed at the Liverpool Magnetic Resonance Imaging Centre (LiMRIC; www.liv.ac.uk/limric), using a Siemens Prisma 3T scanner. Blood/saliva will be extracted from participants at LiMRIC and stored at the Liverpool University Biobank (LUB) freezer room.

### Eligibility criteria

Based on sample size calculations (see below), we will recruit 107 people with a new diagnosis of focal epilepsy and 48 healthy controls. Inclusion and exclusion criteria for patients and controls are outlined below.

#### Inclusion criteria

##### Patients with epilepsy

- Patients who are attending or have attended clinics at WCFT and SRFT who have been diagnosed with focal epilepsy (eg, temporal or frontal lobe epilepsy) by a neurologist.
- Maximum of 3 months since diagnosis.
- Between and including the ages 16–65 years.

##### Healthy controls

- No history of neurological or psychiatric illness or disease.
- Between and including the ages 16–65 years.
- No use of drugs or over four units of alcohol consumed in the preceding 48 hours.

#### Exclusion criteria

##### Patients with epilepsy

- Non-epileptic seizures.
- Single seizures.
- Primary generalised seizures.
- Provoked seizures only (eg, alcohol).
- Known inflammatory neurological condition (specifically multiple sclerosis or sarcoidosis).
- Acute symptomatic seizures (eg, acute brain haemorrhage or brain injury).
- Progressive neurological disease (eg, known brain tumour).
- Previous neurosurgery.
- Concomitant infection.
- Any other significant morbidity (physicians discretion).

##### Healthy controls

- Any neurological disease or illness.
- Drug use or five or more units of alcohol consumed in the preceding 48 hours.

#### MRI criteria

All participants will be examined by a radiographer and will complete a safety checklist that is designed to identify whether a participant has internal bodily metal, which could pose a hazard during MRI scanning. All removable bodily metal will be removed before scanning. Standard MRI exclusion criteria include:

- Internal bodily metal.
- Cardiac pacemaker or defibrillator.
- Cochlear, otologic or ear implant.
- Any implant held in place by a magnet.
- Implanted catheter, clamp, clips, valves or other metal.
- Presence or history of claustrophobia.
- Pregnancy.
- Unremovable bodily piercings or other metal.

### Sample size calculation

Taking into account that roughly 2/3 of patients with NDE are expected to achieve 12 months of remission within 2 years^21 22^ approximately 72 patients with NDE (48/24 patients who achieve/do not achieve remission) and 48 controls are required to detect large effect sizes of 1.2 or above (large effects sizes are supported by our previous findings),^23^ with power 90% and significance level of 0.001. Given the nature of the study and that a panel of biomarkers will be tested, a low significance level has been chosen to control for the false discovery rate (type I error).^24^ In the calculations we have also accounted for the ratio 2:1 for patients with NDE who achieve/do not achieve remission. After taking into account that ~25% patients will present with brain lesions,^25 26^ and considering a potential attrition rate of 10%, a total of 107 patients with a new diagnosis of focal epilepsy will be recruited. Our experience leading multicentre clinical trials in patients with NDE,^27–29^ and considering the inclusion criteria, is that it will take 30 months to recruit this number of patients from the WCFT and SRFT. The proposed sample size will also provide enough power to detect large effect sizes between NDE patients and controls with respect to neuropsychological performance^30^ and therefore make it possible to address Objective 1.

### Recruitment process

**Figure 1:**
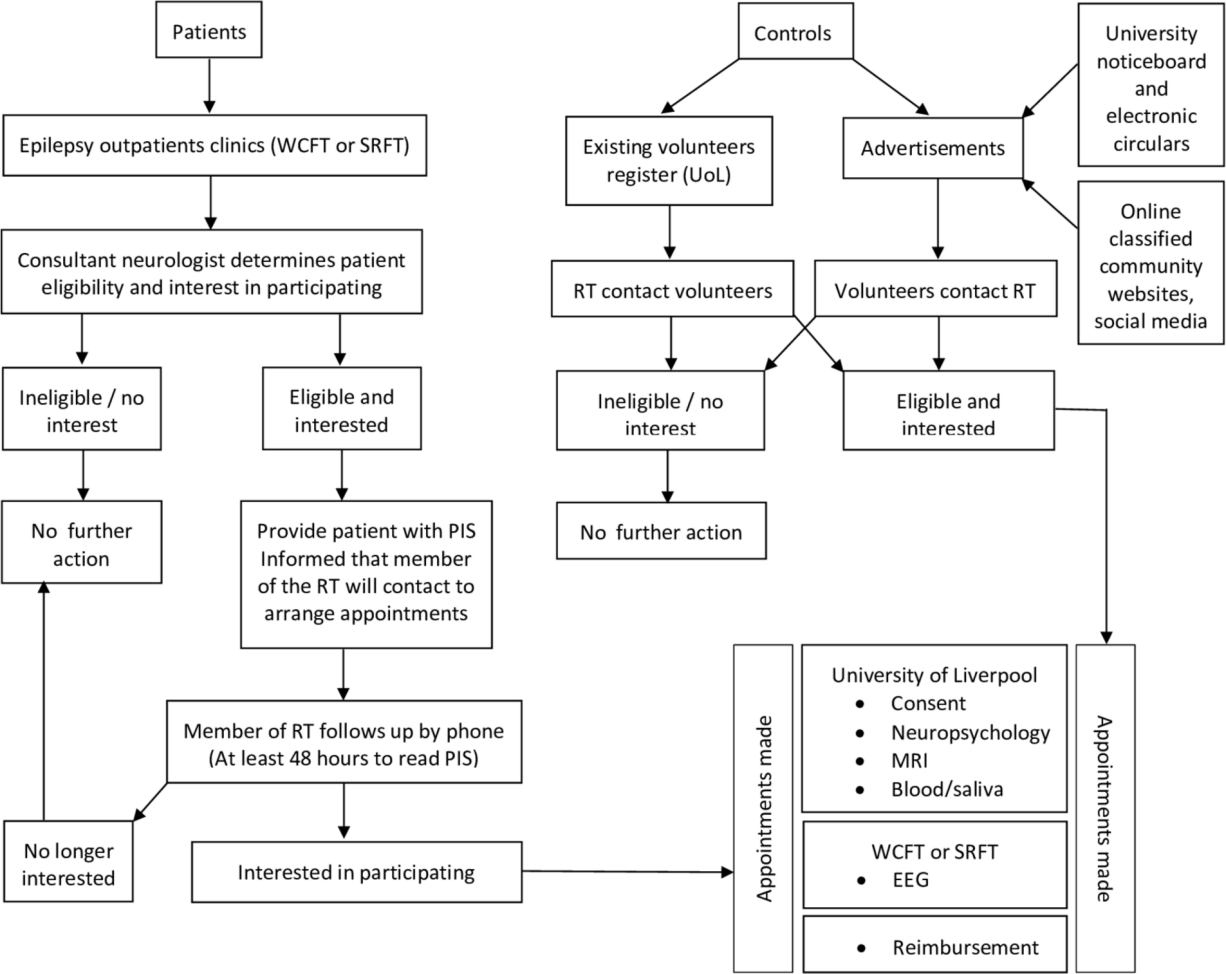
The recruitment process. EEG, electroencephalography; PIS, participant information sheet; RT, research team; SRFT, Salford Royal NHS Foundation Trust; UoL, University of Liverpool; WCFT, Walton Centre Foundation NHS Trust.

A summary of the recruitment process is shown in figure 1. We will recruit participants attending WCFT and SRFT epilepsy clinics according to the aforementioned inclusion criteria. A clinical member of the research team (RT) (ie, consultant neurologist, epilepsy nurse) will enquire whether eligible patients would be interested in participating in this study at the time of consultation in outpatient clinics. If so, the patient will be provided with an information sheet and consent form and allowed at least 48 hours to consider participation. The patient will then be contacted by telephone by a member of the RT to discuss participation, and if the patient would still like to participate, an appointment will be made for the investigations. Patients will bring their signed and dated consent forms with them to their appointment. A member of the RT will confirm consent for each patient.

Healthy controls will be recruited from an existing volunteer register and advertisements placed on UoL notice boards. The recruitment of controls will be age-, sex- and educationally matched. If we struggle to recruit educationally matched controls, we will advertise to the local (Liverpool) community using online classified advertisements and community websites. A member of the RT will determine eligibility and interest of potential controls. Volunteers will be provided with a study information sheet and consent form via email. Eligible volunteers will be given an appointment for investigation. Control volunteers will bring their signed and dated consent forms with them to their appointment. A member of the RT will confirm consent for each control volunteer.

All participants will receive reimbursement of £100 for their participation in this study.

### Participant withdrawal

Participants may withdraw their participation in this study at any time by contacting the RT. If participants withdraw from the study, information that has already been obtained will be kept in minimum personally identifiable format to ensure that their privacy rights are safeguarded.

### Outcomes

The primary treatment outcome variable is seizure outcome 2 years after diagnosis, which is a reliable time point and frequently used marker of pharmacoresistance.^22 31^ Seizure freedom will be defined as a period of no seizures within the preceding 12 months at 2-year outcome, which aligns with current UK driving legislation.^32^ The number and type of seizures experienced since the last follow-up and current medication will be recorded by telephone by an epilepsy specialist nurse using a brief questionnaire adapted from the Standard and New Antiepileptic Drugs (SANAD) II clinical trial. In order to address Objective 1, we require control imaging and cognitive data from healthy participants, which will be compared with corresponding patient data. Based on previous findings,^25 26^ ~25% of patients with NDE recruited are expected to have an identifiable lesion. Although the primary focus of this study will be on patients with MRI-negative NDE, as these represent the large majority of cases, having imaging and neuropsychological data from patients with lesional NDE will allow us to investigate whether the contribution of aberrant brain architecture and function is more significant than gross brain lesions for the prediction of cognitive dysfunction and treatment outcome. In brief, outcomes will consist of statistically significant differences in structural and functional brain connectivity and cognition between patients and controls as well as between patients with and without seizures 2 years after diagnosis.

### Study phases

**Figure 2:**
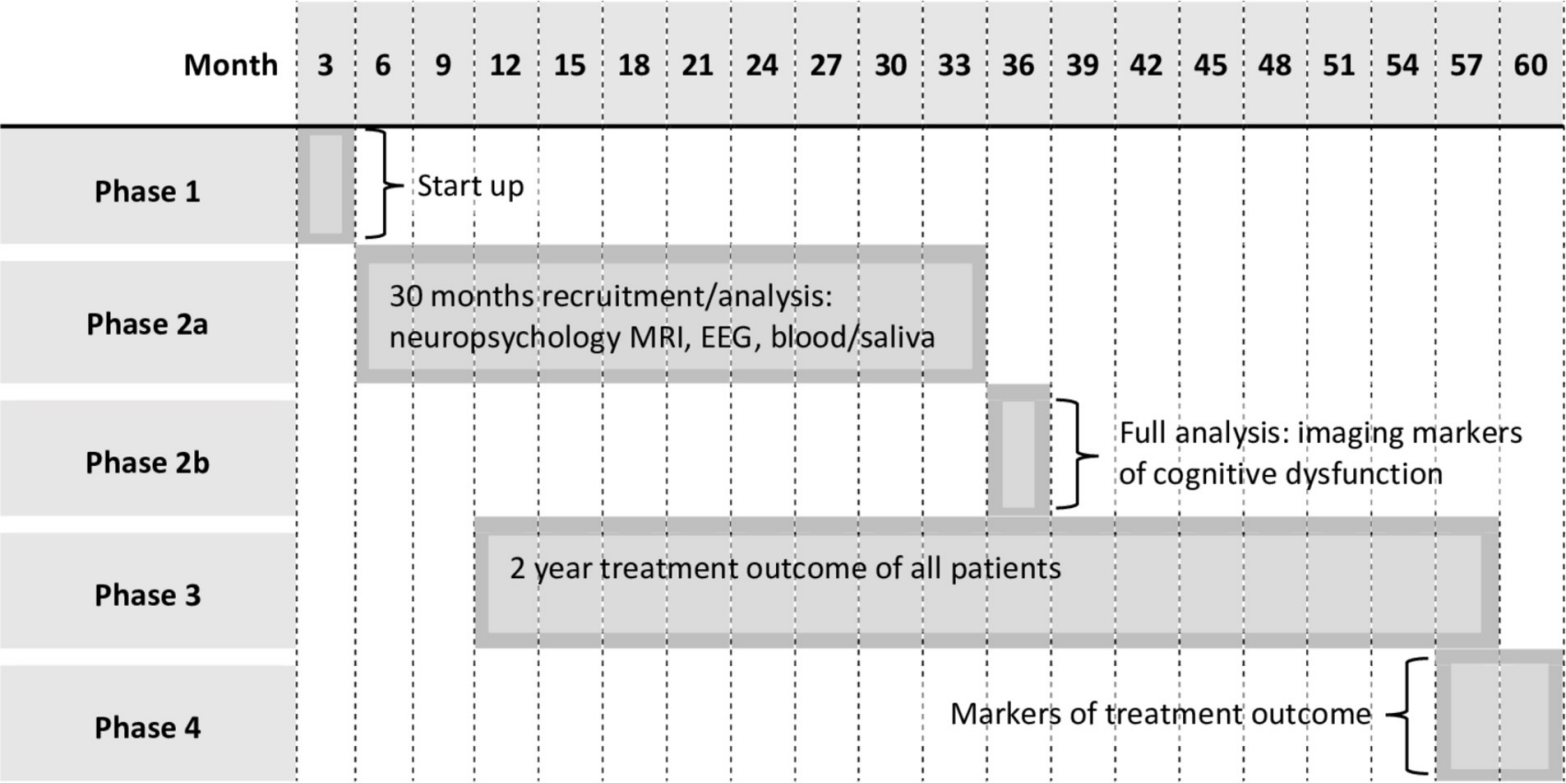
Organisation of the study phases.

The study will last 5 years – from 1 October 2019 to 1 October 2024 – and be split into four phases. Five years are necessary given recruitment and follow-up objectives (Objective 2): we require a recruitment period long enough to recruit a sufficient number of patients with NDE and a follow-up period long enough to establish likely seizure remission/pharmacoresistance. Figure 2 graphically illustrates the organisation of study phases.

#### Phase 1

[Ph1; month 1–3] is an initial 3-month period dedicated to project set-up, optimisation of the MRI protocol and psychologist training for proficient administration of the neuropsychology battery. MRI optimisation will include technical development, MRI scanning of phantoms and human volunteers to ensure the MRI sequences are adequate for the study.

#### Phase 2

[Ph2; month 4–33] is a 30-month period that includes participant recruitment, and MRI, EEG, neuropsychological and blood serum and saliva data acquisition for all recruited participants. The imaging data acquired for all patients and controls will be processed using image analysis techniques throughout Ph2. The final 3 months of Ph2 will be dedicated to the analysis of imaging markers of cognitive dysfunction to address Objective 1 once all imaging and neuropsychological data is collected (Ph2b).

#### Phase 3

[Ph3; month 10–57] is a 48-month patient follow-up period during which time all seizure outcome information will be recorded by telephone by a research nurse at the WCFT. Standardised assessment of patient seizure outcomes will be performed at 6, 12, 18 and 24 months after enrolment into the study. Although the 24-month assessment is the primary outcome time point for this study, we will endeavour to monitor patient status beyond the life of the grant award.

#### Phase 4

[Ph4; month 55–60] is the final 6-month period dedicated to addressing Objectives 2 and 3 when all outcome data are available using multivariate statistics and prognostic modelling.

### Data acquisition

In total, we will perform 155 MRI, EEG, neuropsychological and blood/saliva sample investigations. Neuropsychological, MRI and blood/saliva sample data collection will be performed at the Liverpool Magnetic Resonance Imaging Centre (LiMRIC, Research Technology Building, UoL). The MRI protocol will include clinical sequences for diagnostic appraisal (see below), and a consultant neuroradiologist will review the scans of each participant as per standard clinical protocol. EEG data collection will take place at the WCFT and SRFT. Patients identified at SRFT will be transported from Manchester to Liverpool. A summary of the procedures for each participant is shown of table 1.

**Table 1:** Summary of procedures

Procedure	Location	Duration	Number of examinations
1.Consent	Quiet assessment room, UoL	10 min	1
2.Neuropsychological evaluation	Quiet assessment room, UoL	2 hours, including comfort breaks	1
3.MRI	LiMRIC, UoL	1 hour, including safety examination and set-up	1
4.Blood/saliva extraction	UoL	5 min	1
5.EEG	Neurophysiology, WCFT and SRFT	1 hour, including set-up	1
6.Telephone questionnaire	Home	5 min	4 (6, 12, 18 and 24 months after scans)

EEG, electroencephalography; LiMRIC, Liverpool Magnetic Resonance Imaging Centre; MRI, Magnetic Resonance Imaging; SRFT, Salford Royal NHS Foundation Trust; UoL, University of Liverpool; WCFT, Walton Centre Foundation NHS Trust.

#### LiMRIC

*Consent*. Informed consent will be taken before assessments.

*Neuropsychology*. We will use a computerised neuropsychological battery (lasting up to 2 hours, including comfort breaks) that we have shown to be sensitive to cognitive deficits in people with NDE.^30 33^ These will include components from the Wechsler Memory Scale Fourth Edition (WMS-IV),^34^ Wechsler Adult Intelligence Scale Fourth Edition (WAIS-IV),^33 34^ Delis-Kaplan Executive Function System (D-KEFS),^35 36^ Patient Health Questionnaire 9 (PHQ-9),^37^ Generalised Anxiety Disorder 7 (GAD-7),^38^ The A-B Neuropsychological Assessment Schedule (ABNAS)^39^ and Quality of Life in Epilepsy (QOLIE-31) scale.^40^ More specifically these assessment tools will be used to evaluate:

- Auditory memory through story recall and recall of verbal pairs (WMS-IV).
- Visual memory through reproduction of drawings and recall of designs (WMS-IV).
- Working memory and attention through digit span and arithmetic tasks (WAIS-IV).
- Processing speed through a coding and symbol search task (WAIS-IV).
- Psychomotor speed through a finger tapping and visual reaction time task.
- Executive functioning through verbal fluency and colour-word interference tasks (D-KEFS).
- Mood including depression (PHQ-9) and anxiety (GAD-7).
- Perceived cognitive impairment (ABNAS).
- Quality of life (QOLIE-31).

*MRI scanning*. The MRI protocol will be performed on a 3T Siemens Prisma MRI at LiMRIC and will consist of the following sequences:

- Conventional 2D T2-weighted fast spin echo and fast fluid attenuated inversion recovery scans, for incidental findings screening, and detection of gross pathology (together with localiser 11:00 min).
- 3D T1-weighted MPRAGE (Magnetization-Prepared Rapid Acquisition with Gradient Echo) scan with isotropic voxel size of 1 mm × 1 mm × 1 mm (7:30 min).
- fMRI verbal memory task scan adapted from Sidhu *et al*.^41^: whole brain echo planar imaging (EPI) sequence, with voxel size of 2 mm × 2 mm × 2 mm, Repetition Time (TR)=2.75; 10 concrete nouns shown for 3 s in 10 blocks of 30 s followed by a 15 s baseline period (fixation cross); participants indicate whether each word is pleasant or unpleasant (8:23 min).
- Resting-state fMRI with eyes open with relaxed fixation on projected crosshair, whole brain EPI sequence, with voxel size of 2 mm × 2 mm × 2 mm, TR=2.5 (8:02 min).
- Diffusion kurtosis imaging (DKI) sequence with 60 isotropically distributed gradient directions, three b values (b=0, 1000 and 2000) and maximum voxel size of 2 mm × 2 mm × 2 mm (8:06 min).

*Post-scanning task*. A verbal recognition task of words presented in the fMRI verbal memory task will be completed outside the scanner (<7 min).

*Blood extraction*. Blood will be collected for analysis in lithium-heparin bottles or serum separator tubes (9 mL). A maximum of 72 mL of blood (3×9 mL vials) will be obtained from each participant. Samples will be obtained by a healthcare professional trained in phlebotomy. A standard operating procedure for blood sampling including aseptic technique will be utilised.

*Saliva extraction*. Samples of unstimulated saliva will be collected by soaking a sponge swab in the mouth of each participant until the swab is saturated with saliva. The swab will be inserted into a collection tube.

#### WCFT and SRFT

*EEG*. All participants will undergo a conventional clinical EEG, using 19 channels in 10–20 arrangement. Patients will be scanned in context of standard care in their respective trust (WCFT or SRFT) while controls will be scanned at the WCFT. Participant visiting time will last approximately 1 hour.

### Data analysis

All MRI and EEG analysis techniques are automated and not subject to investigator bias.

#### MRI analysis

The MRI analysis procedures that will be carried out include (but will not be exclusive to):

*Thalamocortical analysis*. Our preliminary (unpublished) data have indicated that patients with NDE have structural changes in the thalamus. We will use DKI approaches to examine thalamic and thalamocortical connectivity. Mean DKI values will be obtained from spatially co-registered regions-of-interest (ROIs) (principally thalamocortical regions) in standard space. We will also apply diffusion^42^ and resting-state fMRI^43^ independent component analysis techniques using FSL’s MELODIC (Multivariate Exploratory Linear Optimized Decomposition into Independent Components) toolbox^44^ (http://fsl.fmrib.ox.ac.uk/fsl/fslwiki/MELODIC) and in-house MATLAB scripts to identify abnormal structural and functional thalamocortical connectivity in patients relative to controls. We will also compare patient neuropsychological and treatment outcome groups using these approaches.

*White matter tracts*. Our recent publications have indicated that analysis of white matter tract diffusion has significance for predicting postsurgical seizure outcome in patients with chronic focal epilepsy^12 16^ and that DKI is more sensitive to tract pathology than diffusion tensor imaging in epilepsy.^45^ As white matter tracts constitute the structural connections within brain networks, we will determine DKI properties along the length of multi-lobar white matter tract bundles, using our recently reported methods.^45 46^

*Large-scale functional networks*. Using our recently described resting-state analysis techniques,^23^ we will identify and analyse features of the major resting-state networks, including the fronto-parietal attentional network, default mode network, salience network and language network. All analyses will be performed using the functional connectivity toolbox.^47^

*Graph theory (Connectome*). The development of whole brain connectomes^48^ from diffusion MRI data has led to successful data-driven approaches to predict surgical responsiveness in patients with refractory focal epilepsy from members of our group.^11 13–15^ Connectome approaches also support the association between postoperative seizure control and thalamocortical connectivity.^14^ Similar methods have been applied to resting-state fMRI data to model functional connectome alterations in chronic focal epilepsy.^49^ As per our recent connectomic studies, whole brain structural connectomes will be generated for each participant using T1-weighted and DKI data. T1-weighted data will be parcellated into multiple ROI (nodes) using Freesurfer software (http://freesurfer.net). Structural connectivity between nodes will be determined using FSL’s diffusion toolbox (http://fsl.fmrib.ox.ac.uk/fsl/fslwiki/FDT) for probabilistic fibre tracking applied to diffusion MRI. Structural connectomes will be generated using the Connectome Mapping Toolkit (http://www.connectome.ch). We will use graph theory to determine global and regional network configuration. Global network ‘small worldness’ will be assessed, representing the ratio between average nodal clustering coefficients and as network efficiency. Regional clustering coefficient, efficiency and centrality will also be calculated for key brain areas associated with seizure onset and propagation such as thalamocortical and limbic networks. We will generate resting-state functional connectomes using a similar approach to structural connectomes. Whereas for structural connectomes edges are represented by diffusion streamlines and kurtosis diffusion scalar metrics, functional connectomes will use fMRI time series correlations between each anatomical ROI to generate a temporal correlation matrix.

*Other network approaches*. Using DKI, T1 and resting-state fMRI data, we will apply network-based statistics to explore network alterations common to subgroups of patients,^50^ and machine/deep learning algorithms to classify individual patient outcomes.^13^

*Verbal memory fMRI*. Following previous work,^41^ we will explore the relationship between memory deficits and neural processing of verbal memory using task-based fMRI. An event-related analysis (FSL-FEAT) will be used to examine the neural correlates of successful subsequent memory formation, comparing memory encoding networks between patients and controls, as well as between seizure-free and drug-resistant epilepsy patients. Between-group ROI comparisons in temporal and extra-temporal regions will also be made with neuropsychological variables included as covariates into a general linear model. We hypothesise that altered neural processing of verbal memory will be observed in patients with memory-related deficits and that network organisation differences centred around temporal regions will be observed in patients with drug-resistant epilepsy.

#### EEG analysis

Resting-state EEG activity will be identified by a trained clinical EEG technician. Nodes in EEG networks will be defined as electrodes, and a range of measures of interdependence between electrodes will be explored. We will apply computer models of network dynamics to resting-state EEG data.^10^ In order to address Objective 1 and Objective 2, we will analyse EEG network dynamics by mirroring the approach we will take with MRI (thalamocortical and connectome analysis); first, we will focus on thalamocortical physiological alterations by source-localising activity within thalamic and cortical regions and determine connectivity between regions using dynamic causal modelling.^51^ Second, we will reconstruct resting-state EEG connectomes consistent with the resting-state functional MRI approach and determine network-based physiological differences between groups. We will also explore the inter-relation between EEG and MRI determined connectivity by performing DKI tractography seeded from nodes identified as aberrantly connected using EEG to determine whether abnormal physiological connectivity is related to abnormal structural connectivity. This approach has been adopted in patients with refractory epilepsy who underwent stereoelectroencephalography.^52^

#### Blood and saliva sample analysis

In the laboratory, blood samples will be centrifuged within 15 min of collection or stored overnight at 4°C for centrifugation the following day. 250 µl aliquots will then be transferred to appropriate tubes and stored at approximately −80°C prior to bioanalysis. Saliva samples will be collected into an Eppendorf tube by squeezing the saturated swab using a syringe. The sample will be stored at −80°C freezer until assay. Blood and saliva samples will be analysed for inflammatory markers, HMGB1 and brain-specific markers including microRNA. Inflammatory marker and HMGB1 expression analysis will be undertaken by ELISA and HMGB1 quantification will be made by liquid chromatography and mass spectrometry. Blood/saliva samples will be stored in the LUB freezer room, which is housed in the Research Technology Building with LiMRIC.

#### Statistical analysis

We will explore the discriminatory effect of imaging biomarkers when taking into account their correlation structure and develop predictive models.

##### Cognitive dysfunction

Multivariate discriminant techniques will be used to identify structural/physiological brain measures that significantly differ between controls, patients with NDE who are cognitively normal and patients with NDE who are cognitively impaired (where impairment is defined by patient performance lower than two SD^53^ than that of the group of controls on respective neuropsychological tasks). We will also investigate the relationship between patient cognitive performance and imaging measures of architecture and network connectivity using multivariate regression analyses.

##### Treatment outcome

Multivariate data techniques will be used to determine imaging measures that significantly differ between controls, patients who achieve remission over 12 months within 2 years and patients who do not achieve remission within the same time period. Embedded multivariate techniques such as least absolute shrinkage and selection operator and the support vector machines approaches (eg, with radial and polynomial basis function kernels) will be applied to identify the panel of biomarkers with optimal discriminatory ability. For this process we will also consider patient demographic and clinical data, and presence of brain lesion. The variable selection algorithms will take into account the correlation between the variables, as well as variance differences across groups. To minimise the effect of possible over-fitting, penalty terms will be embedded in the variable selection algorithm to take into account model complexity. We will develop prognostic models for the assessment of outcome at 2 years using multivariate discriminant analysis.^54^ Cross-validation and bootstrap techniques will be applied when appropriate.

## Ethics and dissemination

### Ethical approval

The PI will ensure that the study is conducted in full accordance with approved protocols and that agreed modifications are disseminated to all relevant parties.

### Confidentiality

Procedures for handling, processing, storage and destruction of your data are compliant with the Data Protection Act 1998. All EEG and MRI data will be anonymised prior to being exported from the WCFT, SRFT and LiMRIC, respectively. Personal information will not be identifiable from the imaging data. Names will be replaced with study ID numbers (EPINET001, EPINET002, etc), which can be backtracked to participant details using a key located at LiMRIC and only accessible to the primary care team. Storage eppendorfs will be labelled with the unique identifier only with no other patient information. Similarly, all electronic neuropsychological data will be associated with study ID numbers. All documents associated with the study will be stored securely and only accessible by research staff and authorised personnel. Participant recruitment data will be monitored through study adoption by the National Institute for Health Research (NIHR) portfolio.

### Informed consent

All participants will be provided with a research information pack describing the nature and goals of the research, and study consent form, which must be completed, signed and dated. We will not recruit participants who lack capacity to provide informed consent (eg, those with intellectual disability or dementia). Study consent forms will be retained and filed in a locked cupboard in the office of the PI. Information packs and consent forms will be given to people with epilepsy by a research nurse immediately after diagnosis in outpatient clinics. Information packs will be sent to healthy control volunteers via email or post. All participants will be given consent forms to complete at their first scanning appointment bring consent forms with them to their appointment for MRI and EEG scanning. The RT will the take the participant through the information sheet and consent form, explaining any aspects of the study that the participant is unclear about. Patients and controls will have as long as they require to consider their decision to volunteer for the research or not. The investigators contact details will be provided in the information pack.

### Potential benefits and risks

There are no direct benefits to the participant. However, participation may lead to improved understanding of the aetiology, development and prognosis of epilepsy in the future.

MRI is considered a safe technique and scanning environment. The MRI system produces a high magnetic field, and it is necessary for the participants to remove all ferrometallic objects from their person before entering the scanning room. As per routine protocol, clinical members of staff screen participants for their suitability for scanning and fully debrief them after scanning. The MRI scanner is a noisy and confined environment, which may cause the participant to feel slight discomfort and claustrophobia. During scanning the subject will be monitored from the MRI control room by clinical staff and their heart rate continuously monitored. If participants feel discomfort, scanning can be discontinued by pressing a distress button that the participant will be given before entering the scanner.

As indicated in the study information pack, scans will be reviewed to make sure that there is no brain pathology. Any MRI incidental finding will be reported to a consultant neuroradiologist. If the MRI finding is deemed to warrant further investigation, the participant’s General Practitioner (GP) may be contacted. The percentage of unanticipated clinically serious brain abnormalities in healthy people is extremely low.

Venipuncture poses minimal risk of bruising or bleeding. All efforts will be made to minimise the risk of infection; appropriate training in infection control will be undertaken by all healthcare professionals.

There are no potential risks of EEG recordings. Neuropsychological evaluation could potentially lead to participant fatigue, frustration or emotional disturbance. To obviate this, we will provide each participant with sufficient resting time between each assessment.

### Patient and public involvement

No patients were directly involved in the design of this study. However, all participants will be fully debriefed regarding the goals and design of the research and will have the option of receiving a letter with a brief summary of the results at the end of the study.

### Dissemination

We aim to produce high-impact peer-reviewed publications of the results of the study and present findings at national and international conferences, with exclusive access to the final study data set for a period of 6 years. We will target epilepsy (eg, European Congress for Epileptology, International Epilepsy Congress, International League Against Epilepsy UK Chapter, American Epilepsy Society) and neuroimaging (eg, The Organisation of Human Brain Mapping, International Society for Magnetic Resonance in Medicine) conferences. The investigators will be involved in preparing manuscripts drafts, abstracts, press releases among any other publications arising from the study and will acknowledge that the study was funded by the MRC. For each publication, only members of the RT who made a significant intellectual contribution to each piece of work will be considered as an author. This is in line with journal protocol. All authors share responsibility for the contents of the submitted manuscript.

## Supplementary Material

Reviewer comments

Author's manuscript
