# Supplementary material for: Investigating imaging network markers of cognitive dysfunction and pharmacoresistance in newly diagnosed epilepsy: a protocol for an observational cohort study in the UK
Source: BMJ Open. 2019 Oct 16;9(10):e034347. doi: 10.1136/bmjopen-2019-034347 (PMC6797398; doi:10.1136/bmjopen-2019-034347)
Supplement: Author's manuscript [file bmjopen-2019-034347.draft_revisions.pdf]

BMJ Open is committed to open peer review. As part of this commitment we make the peer review history of every article we publish publicly available.

When an article is published we post the peer reviewers' comments and the authors' responses online. We also post the versions of the paper that were used during peer review. These are the versions that the peer review comments apply to.

The versions of the paper that follow are the versions that were submitted during the peer review process. They are not the versions of record or the final published versions. They should not be cited or distributed as the published version of this manuscript.

BMJ Open is an open access journal and the full, final, typeset and author-corrected version of record of the manuscript is available on our site with no access controls, subscription charges or pay-per-view fees (http://bmjopen.bmj.com).

If you have any questions on BMJ Open's open peer review process please email info.bmjopen@bmj.com

BMJ Open

A protocol for an observational cohort study investigating imaging network markers of cognitive dysfunction and pharmacoresistance in newly diagnosed epilepsy.

Journal:	*BMJ Open*
Manuscript ID	bmjopen-2019-034347
Article Type:	Protocol
Date Submitted by the Author:	16-Sep-2019

Complete List of Authors:	de Bézenac, Christophe; University of Liverpool, Molecular and Clinical Pharmacology, Institute of Translational Medicine<br>Garcia-Finana, Marta; University of Liverpool, Biostatistics<br>Baker, Gus; University of Liverpool Faculty of Health and Life Sciences, Molecular and Clinical Pharmacology<br>Moore, Perry; Salford Royal NHS Foundation Trust<br>Leek, Nicola; University of Liverpool, 1. Department of Molecular and Clinical Pharmacology, Institute of Translational Medicine<br>Mohanraj, Rajiv; Salford Royal NHS Foundation Trust<br>Bonilha, Leonardo ; Medical University of South Carolina, Department of Neurology<br>Richardson, Mark; King's College London, Institute of Psychiatry, Psychology & Neuroscience<br>Marson, Anthony; University of Liverpool, Molecular and Clinical Pharmacology<br>Keller, Simon; University of Liverpool, Department of Molecular and Clinical Pharmacology, Institute of Translational Medicine

Keywords:	Epilepsy < NEUROLOGY, Brain connectivity, Neuropsychology, Inflammation, Newly diagnosed

SCHOLARONE™
Manuscripts

A protocol for an observational cohort study investigating imaging network markers of cognitive dysfunction and pharmacoresistance in newly diagnosed epilepsy.

Christophe de Bézenac[1], Marta García-Fiñana[2], Gus A. Baker[1,3], Perry Moore[4], Nicola Leek[1], Rajiv Mohanraj[4], Leonardo Bonilha[5], Mark P. Richardson[6], Anthony G. Marson[1,3] and Simon S. Keller[1,3]

1. Department of Molecular and Clinical Pharmacology, Institute of Translational Medicine, University of Liverpool, Liverpool, L69 3BX, United Kingdom.
2. Department of Biostatistics, Institute of Translational Medicine, University of Liverpool, Liverpool, L69 3BX, United Kingdom.
3. The Walton Centre NHS Foundation Trust, Lower Lane, Liverpool, L9 7LJ, United Kingdom.
4. Salford Royal NHS Foundation Trust, Salford M6 8HD, United Kingdom.
5. Department of Neurology, Medical University of South Carolina, Charleston, USA
6. Department of Basic and Clinical Neuroscience, Institute of Psychiatry, Psychology and Neuroscience, King's College London, London, UK

Christophe de Bézenac: cedb@liverpool.ac.uk; +44 (0)151 529 5943 (corresponding author)
Marta García-Fiñana: martaf@liverpool.ac.uk; +44 (0)151 794 9755.
Gus Barker: gus@liverpool.ac.uk; +44 (0)151 529 5948
Perry Moore: Perry.Moore@srft.nhs.uk; +44 (0)161 789 7373
Nicola Leek: nleek@liverpool.ac.uk; +44 (0)151 529 5943
Rajiv Mohanraj: Rajiv.Mohanraj@srft.nhs.uk; +44 (0)161 206 4626
Leonardo Bonilha: bonilha@musc.edu; +1 843 876 8311
Mark Richardson: mark.richardson@kcl.ac.uk; +44 (0)207 848 5429
Anthony Marson: marjon01@liverpool.ac.uk; +44 (0)151 529 5705
Simon Keller: Simon.keller@liverpool.ac.uk; +44 (0)151 529 5943

Correspondence to
Christophe de Bézenac,
Liverpool Magnetic Resonance Imaging Centre, University of Liverpool, Pembroke Place, Liverpool, L69 3GE UK
cedb@liverpool.ac.uk;
+44 (0)151 529 5943

For peer review only - http://bmjopen.bmj.com/site/about/guidelines.xhtml

ABSTRACT

Introduction: Epilepsy is one of the most common serious brain disorders, characterised by seizures that severely affect a person's quality of life and, frequently, their cognitive and mental health. Although most existing work has examined chronic epilepsy, newly diagnosed patients presents a unique opportunity to understand the underlying biology of epilepsy and predict effective treatment pathways. The objective of this prospective cohort study is to examine whether cognitive dysfunction is associated with measurable brain architectural and connectivity impairments at diagnosis and whether the outcome of antiepileptic drug treatment can be predicted using these measures.

Methods and analysis: 107 patients with newly diagnosed focal epilepsy from two NHS Trusts and 48 healthy controls (aged 16 to 65 years) will be recruited over a period of 30 months. Baseline assessments will include neuropsychological evaluation, structural and functional MRI, EEG, and a blood and saliva sample. Patients will be followed up every 6 months for a 24-month period to assess treatment outcomes. Connectivity- and network-based analyses of EEG and MRI data will be carried out and examined in relation to neuropsychological evaluation and patient treatment outcomes. Patient outcomes will also be investigated with respect to analysis of molecular isoforms of high mobility group box-1 (HMGB1) from blood and saliva samples.

Ethics and dissemination: This study was approved by the North West, Liverpool East Research Ethics Committee (19/NW/0384) and funded by a Medical Research Council research grant (MR/S00355X/1). Findings will be presented at national and international meetings and conferences and published in peer-reviewed journals.

Trial registration number: IRAS Project ID 260623; Protocol Version 6; Pre-results.

Keywords: Newly diagnosed, Epilepsy, Brain connectivity, Neuropsychology, Inflammation

ARTICLE SUMMARY

Strengths and limitations of this study

- This will be the first study to prospectively investigate brain structural and physiological architecture and connectivity in adults with a new diagnosis of focal epilepsy
- The study is expected to provide insights into the biology underlying cognitive dysfunction in the early stages of human epilepsy, and to lead to the development of prognostic markers of future pharmacoresistance.
- Expected recruitment has been based on records of past diagnosis at recruitment sites and while the study is expected to recruit well, unexpected under-recruitment is possible and would be a barrier to timely completion.
- A second potential limitation of this study is the potential for participant attrition and loss of patient follow up at multiple points over 24 months; missing data could impact on the validity of study conclusions.

INTRODUCTION

Background and rationale

Epilepsy is one of the most common serious brain disorders; every day in the UK, 87 people are diagnosed with epilepsy, affecting over 600,000 people.[1] The condition is characterised by devastating seizures that severely impact on a person's quality of life. Epilepsy frequently affects a person's cognitive and mental health,[2] and the disorder contributes to elevated propensity for depression, suicide and sudden and unexpected death compared to the general population.[3,4] Despite this, research into epilepsy has been grossly underfunded compared to other medical conditions of similar economic, social and personal impact.[5] The vast majority of existing work in human studies has been performed in chronic epilepsy. Newly diagnosed epilepsy (NDE) is only rarely studied despite representing a key point in time to understand the underlying biology of the disorder in the absence of confounds including anti-epileptic drugs (AEDs) and long term seizure effects.[6] It is important to understand the reasons why people with epilepsy experience cognitive problems and seizures after treatment using safe imaging technologies from the earliest time point of the disorder. If we can understand these reasons in the early stages of epilepsy, we may be able to predict which patients will continue to experience seizures despite standard drug therapy. Patients who will not respond to drug therapy could potentially be offered alternative or adjunctive treatments, saving time, cost, and the experience of undesirable side effects of certain AEDs.

For peer review only

For peer review only - http://bmjopen.bmj.com/site/about/guidelines.xhtml

MRI and EEG are routinely used to assess people with epilepsy. However, the application of these brain imaging techniques in the context of standard care cannot determine why some patients have cognitive problems and why others do not, and why some patients do not respond to AED therapy while others do. A new direction of brain imaging is therefore required; preferably one that can be incorporated into the standard clinical evaluation of patients. In patients with longstanding epilepsy the study of brain connectivity and networks (how different regions of the brain work together by virtue of their connectivity) using MRI and EEG has recently provided valuable insights into how the brain is structurally and physiologically altered in epilepsy.[7,8] There is growing evidence that aberrant network dynamics are a key part of the underlying mechanisms of focal and generalised epilepsies.[9] State-of-the-art quantitative structural (e.g. diffusion MRI and tractography approaches), functional (e.g. resting-state functional MRI), and physiological (e.g. EEG) imaging techniques have provided a novel way of automatically distinguishing longstanding epilepsy patients from healthy controls,[10] and predicting postoperative treatment outcome in severe epilepsy.[11-16] We propose that these approaches will provide new explanations for the causes of cognitive problems and future treatment outcome from the beginning of a patient's life with epilepsy.

Furthermore, mechanistic blood and saliva biomarkers could greatly enhance drug discovery by providing novel therapeutic targets and enrich trial populations, facilitating early surgical evaluation in drug-resistance. We have soon-to-be-published data suggesting that molecular isoforms of high mobility group box-1 (HMGB1) – a protein critically involved in the initiation of the inflammatory cascade in epilepsy –[17] have potential as a prognostic biomarker. The acetylated, disulfide form of HMGB1, which triggers pro-inflammatory cytokine release via toll-like receptor 4, has shown pathological effects in pre-clinical models of seizures.[18] In a parallel running study, we are currently studying people with longstanding epilepsy using MRI and blood serum markers of HMGB1 (Short title: "MRI of inflammation in epilepsy"; IRAS project ID 220138; REC reference 17/NW/0342, Northwest-Liverpool).

This observational cohort study will be the first to prospectively investigate brain structural and physiological architecture and connectivity in adults with NDE with overarching goals to: (1) understand the neural basis of cognitive impairment; and (2) identify why and in whom seizures persist despite AED therapy. We will recruit adults with a new diagnosis of focal epilepsy and perform cognitive assessment and sophisticated analysis of MRI and EEG data. At the time of scanning and neuropsychological evaluation (baseline), all participants will additionally have blood and saliva extracted. Patients will be followed up longitudinally to determine their response to AED therapy. MRI/EEG data will be used to identify the neural correlates of cognitive impairment and to predict treatment outcome. Data generated from extracted blood and saliva samples will also be used to predict treatment outcome. To remain consistent with our ongoing work that investigates the correlation between MRI data in HMGB1 in people with epilepsy, we will use an identical approach of data acquisition and analysis. This work will be performed in an environment with demonstrated excellence in the care of people with epilepsy, recruitment of adults with NDE into clinical trials, and expertise in MRI, EEG, neuropsychological and blood serum analysis. The research objectives of the proposed work directly address internationally agreed research priorities in epilepsy, with potential to provide significant insights into the epilepsy phenotype and to generate clinically meaningful non-invasive markers of treatment outcome.[19,20]

Study objectives and design

The goal of the proposed research is to perform the first prospective multi-modal imaging investigation of brain architecture and connectivity in adults with a new diagnosis of focal epilepsy. The project aims to provide new insights into the biology underlying cognitive dysfunction in the early stages of human epilepsy and develop prognostic markers of future pharmacoresistance. The research will take place in context of a collaborative research and clinical environment that has demonstrated excellence in the recruitment and study of patients with newly diagnosed epilepsy. The three main objectives are outlined below.

Objective 1

The primary objective is to determine the cognitive phenotype associated with newly diagnosed epilepsy and whether cognitive dysfunction is associated with measurable brain architectural and connectivity impairments at diagnosis. We expect that patients will be cognitively impaired in the domains of memory, sustained attention and executive function; this impairment will be reflected in pathological alterations to structural and functional neural networks and responses to a verbal memory task computed from f/MRI and EEG.

For peer review only - http://bmjopen.bmj.com/site/about/guidelines.xhtml

Objective 2

A secondary objective is to determine whether AED treatment outcome can be predicted using multi-modal imaging measures of brain architecture and connectivity at the point of epilepsy diagnosis. We expect that architectural and physiological alterations within local and networked brain regions can predict patient response to pharmacological therapy at diagnosis.

Objective 3

We will determine whether blood serum and saliva derived measures of inflammation can predict AED treatment outcome in patients with newly diagnosed epilepsy and examine relationships between molecular isoforms of HMGB1 and MRI, EEG and neuropsychological data.

METHODS AND ANALYSIS

Study environment

Research will be carried out by the Epilepsy Research Group within the Institute of Translational Medicine (ITM), University of Liverpool (UoL). The group is closely affiliated with the Walton Centre (WCFT) Foundation NHS Trust from where patients will be recruited, alongside the Salford Royal NHS Foundation Trust (SRFT). Both WCFT and SRFT will acquire patient EEG data in context of standard clinical care; EEG data for healthy controls will be acquired at the WCFT. MRI acquisition will be performed at the Liverpool Magnetic Resonance Imaging Centre (LiMRIC; www.liv.ac.uk/limric), using a Siemens Prisma 3T scanner. Blood/saliva will be extracted from participants at LiMRC and stored at the Liverpool University Biobank freezer room.

Eligibility criteria

Based on sample size calculations (see below), we will recruit 107 people with a new diagnosis of focal epilepsy and 48 healthy controls. Inclusion and exclusion criteria for patients and controls are outlined below.

Inclusion Criteria

Patients with epilepsy

- Patients who are attending or have attended clinics at WCFT and SRFT who have been diagnosed with focal epilepsy (e.g. temporal or frontal lobe epilepsy) by a neurologist
- Maximum of three months since diagnosis
- Between and including the ages 16-65 years

Healthy controls

- No history of neurological or psychiatric illness or disease
- Between and including the ages 16-65 years
- No use of drugs or over four units of alcohol consumed in the preceding 48 hours

Exclusion Criteria

Patients with epilepsy

- Non-epileptic seizures
- Single seizures
- Primary generalised seizures
- Provoked seizures only (e.g. alcohol)

For peer review only - http://bmjopen.bmj.com/site/about/guidelines.xhtml

- Known inflammatory neurological condition (specifically multiple sclerosis or sarcoidosis)
- Acute symptomatic seizures (e.g. acute brain haemorrhage or brain injury)
- Progressive neurological disease (e.g. known brain tumour)
- Previous neurosurgery
- Concomitant infection
- Any other significant morbidity (physicians discretion)

Healthy controls

- Any neurological disease or illness
- Drug use or five or more units of alcohol consumed in the preceding 48 hours

MRI Criteria

All participants will be examined by a radiographer and will complete a safety checklist that is designed to identify whether a participant has internal bodily metal, which could pose a hazard during MRI scanning. All removable bodily metal will be removed before scanning. Standard MRI exclusion criteria include:

- Internal bodily metal, including
- Cardiac pacemaker or defibrillator
- Cochlear, otologic, or ear implant
- Any implant held in place by a magnet
- Implanted catheter, clamp, clips, valves, or other metal
- Presence or history of claustrophobia
- Pregnancy
- Unremovable bodily piercings or other metal

Sample size calculation

Taking into account that roughly 2/3 of patients with NDE are expected to achieve 12 months of remission within two years [21,22] approximately 72 patients with NDE (48/24 patients who achieve/do not achieve remission) and 48 controls are required to detect large effect sizes of 1.2 or above (large effects sizes are supported by our previous findings)[23], with power 90% and significance level of 0.001. Given the nature of the study and that a panel of biomarkers will be tested, a low significance level has been chosen to control for the false discovery rate (type I error) [24]. In the calculations we have also accounted for the ratio 2:1 for patients with NDE who achieve/do not achieve remission. After taking into account that ~25% patients will present with brain lesions,[25,26] and considering a potential attrition rate of 10%, a total of 107 patients with a new diagnosis of focal epilepsy will be recruited. Our experience leading multicentre clinical trials in patients with NDE,[27-29] and considering the inclusion criteria, is that it will take 30 months to recruit this number of patients from the WCFT and SRFT. The proposed sample size will also provide enough power to detect large effect sizes between NDE patients and controls with respect to neuropsychological performance [30] and therefore make it possible to address Objective 1.

Recruitment process

A summary of the recruitment process is shown in Figure 1. We will recruit participants attending WCFT and SRFT epilepsy clinics according to the aforementioned inclusion criteria. A clinical member of the research team (i.e. consultant neurologist, epilepsy nurse) will enquire whether eligible patients would be interested in participating in this study at the time of consultation in outpatient clinics. If so, the patient will be provided with an information sheet and consent form and allowed at least 48 hours to consider participation. The patient will then be contacted by telephone by a member of the research team (RT) to discuss

For peer review only - http://bmjopen.bmj.com/site/about/guidelines.xhtml

participation, and if the patient would still like to participate, an appointment will be made for the investigations. Patients will bring their signed and dated consent forms with them to their appointment. A member of the RT will confirm consent for each patient.

Healthy controls will be recruited from an existing volunteer register and advertisements placed on UoL notice boards. The recruitment of controls will be age-, sex- and educationally-matched. If we struggle to recruit educationally-matched controls, we will advertise to the local (Liverpool) community using online classified advertisements and community websites. A member of the RT will determine eligibility and interest of potential controls. Volunteers will be provided with a study information sheet and consent form via email. Eligible volunteers will be given an appointment for investigation. Control volunteers will bring their signed and dated consent forms with them to their appointment. A member of the RT will confirm consent for each control volunteer.

All participants will receive reimbursement of £100 for their participation in this study.

[Figure 1 about here.]

Participant withdrawal

Participants may withdraw their participation in this study at any time by contacting the RT. If participants withdraw from the study, information that has already been obtained will be kept in minimum personally-identifiable format to ensure that their privacy rights are safeguarded.

Outcomes

The primary treatment outcome variable is seizure outcome two years after diagnosis, which is a reliable time point and frequently used marker of pharmacoresistance.[31,32] Seizure freedom will be defined as a period of no seizures within the preceding 12 months at 2-year outcome, which aligns with current UK driving legislation.[33] The number and type of seizures experienced since the last follow up and current medication will be recorded by telephone by an epilepsy specialist nurse using a brief questionnaire adapted from the SANAD II clinical trial. In order to address Objective 1, we require control imaging and cognitive data from healthy participants, which will be compared with corresponding patient data. Based on previous findings,[25,26] ~25% of patients with NDE recruited are expected to have an identifiable lesion. Although the primary focus of this study will be on patients with MRI-negative NDE, as these represent the large majority of cases, having imaging and neuropsychological data from patients with lesional NDE will allow us to investigate whether the contribution of aberrant brain architecture and function is more significant than gross brain lesions for the prediction of cognitive dysfunction and treatment outcome. In brief, outcomes will consist of statistically significant differences in structural and functional brain connectivity and cognition between patients and controls as well as between patients with and without seizures two years after diagnosis.

Study phases

The study will last five years and be split into four phases. Five years are necessary given recruitment and follow-up objectives (Objective 2): we require a recruitment period long enough to recruit a sufficient number of patients with NDE and a follow up period long enough to establish likely seizure remission/pharmacoresistance. Figure 2 graphically illustrates the organisation of study phases.

[Figure 2 about here.]

Phase 1

[Ph1; month 1-3] is an initial 3-month period dedicated to project set-up, optimisation of the MRI protocol and psychologist training for proficient administration of the neuropsychology battery. MRI optimisation will include technical development MRI scanning of phantoms and human volunteers to ensure the MRI sequences are adequate for the study.

Phase 2

[Ph2; month 4-33] is a 30-month period that includes participant recruitment, and MRI, EEG, neuropsychological and blood serum and saliva data acquisition for all recruited participants. The imaging data acquired for all patients and controls will be processed

For peer review only - http://bmjopen.bmj.com/site/about/guidelines.xhtml

using image analysis techniques throughout Ph2. The final three months of Ph2 will be dedicated to the analysis of imaging markers of cognitive dysfunction to address Objective 1 once all imaging and neuropsychological data is collected (Ph2b).

Phase 3

[Ph3; month 10-57] is a 48-month patient follow-up period during which time all seizure outcome information will be recorded by telephone by a research nurse at the WCFT. Standardised assessment of patient seizure outcomes will be performed at 6, 12, 18 and 24 months after enrolment into the study. Although the 24-month assessment is the primary outcome time point for this study, we will endeavour to monitor patient status beyond the life of the grant award.

Phase 4

[Ph4; month 55-60] is the final 6-month period dedicated to addressing Objective 2 and Objective 3 when all outcome data is available using multivariate statistics and prognostic modelling.

Data acquisition

In total, we will perform 155 MRI, EEG, neuropsychological and blood/saliva sample investigations. Neuropsychological, MRI and blood/saliva sample data collection will be performed at LiMRC (Liverpool Magnetic Resonance Imaging Centre, Research Technology Building, UoL). The MRI protocol will include clinical sequences for diagnostic appraisal (see below), and a consultant neuroradiologist will review the scans of each participant as per standard clinical protocol. EEG data collection will take place at the WCFT and SRFT. Patients identified at SRFT will be transported from Manchester to Liverpool. A summary of the procedures for each participant is shown of Table 1.

LiMRC

Consent. Informed consent will be taken before assessments.

Neuropsychology. We will use a computerised neuropsychological battery (lasting up to two hours, including comfort breaks) that we have shown to be sensitive to cognitive deficits in people with NDE.[30,34] These will include components from the Wechsler Memory Scale Fourth Edition (WMS-IV),[35] Wechsler Adult Intelligence Scale Fourth Edition (WAIS-IV),[34,35] Delis-Kaplan Executive Function System (D-KEFS),[36,37] Patient Health Questionnaire 9 (PHQ-9),[38] Generalised Anxiety Disorder 7 (GAD-7),[39] The A-B Neuropsychological Assessment Schedule (ABNAS),[40] and Quality Of Life In Epilepsy (QOLIE-31) scale.[41] More specifically these assessment tools will be used to evaluate:

1. Auditory memory through story recall and recall of verbal pairs (WMS-IV)
2. Visual memory through reproduction of drawings and recall of designs (WMS-IV)
3. Working memory and attention through digit span and arithmetic tasks (WAIS-IV)
4. Processing speed through a coding and symbol search task (WAIS-IV)
5. Psychomotor speed through a finger tapping and visual reaction time task
6. Executive functioning through verbal fluency and colour-word interference tasks (D-KEFS)
7. Mood including depression (PHQ-9) and anxiety (GAD-7)
8. Perceived cognitive impairment (ABNAS)
9. Quality of life (QOLIE-31)

MRI scanning. The MRI protocol will be performed on a 3 T Siemens Prisma MRI at LiMRIC and will consist of the following sequences:

1. Conventional 2D T2-weighted fast spin echo and fast Fluid Attenuated Inversion Recovery scans, for incidental findings screening, and detection of gross pathology (together with localizer 11:00 minutes)
2. 3D T1-weighted MPRAGE scan with isotropic voxel size of 1 mm x 1 mm x 1mm (7:30 minutes)

For peer review only - http://bmjopen.bmj.com/site/about/guidelines.xhtml

3. fMRI verbal memory task scan adapted from Sidhu et al.[42]: whole brain echo planar imaging (EPI) sequence, with voxel size of 2 mm x 2 mm x 2mm, TR = 2.75; 10 concrete nouns shown for 3 seconds in 10 blocks of 30 seconds followed by a 15 second baseline period (fixation cross); participants indicate whether each word is pleasant or unpleasant (8:23 minutes)
4. Resting-state fMRI with eyes open with relaxed fixation on projected crosshair, whole brain echo planar imaging (EPI) sequence, with voxel size of 2 mm x 2 mm x 2mm, TR=2.5 (8:02 minutes)
5. Diffusion kurtosis imaging (DKI) sequence with 60 isotropically distributed gradient directions, three b values (b=0, 1000 and 2000) and maximum voxel size of 2 mm x 2 mm x 2mm (8:06 minutes)

Post scanning task. A verbal recognition task of words presented in the fMRI verbal memory task will be completed outside the scanner (<7 minutes).

Blood extraction. Blood will be collected for analysis in a Lithium- Heparin bottles or serum separator tubes (9mls). A maximum of 72 milliliters of blood (3 x 9ml vials) will be obtained from each participant. Samples will be obtained by a healthcare professional trained in phlebotomy. A standard operating procedure for blood sampling including aseptic technique will be utilised.

Saliva extraction. Samples of unstimulated saliva will be collected by soaking a sponge swab in the mouth of each participant until the swab is saturated with saliva. The swab will be inserted into a collection tube.

WCFT and SRFT

EEG. All participants will undergo a conventional clinical EEG, using 19 channels in 10-20 arrangement. Patients will be scanned in context of standard care in their respective trust (WCFT or SRFT) while controls will be scanned at the WCFT. Participant visiting time will last approximately 1 hour.

Data analysis

All MRI and EEG analysis techniques are automated and not subject to investigator bias.

MRI analysis

The MRI analysis procedures that will be carried out include (but will not be exclusive to):

Thalamocortical analysis. Our preliminary (unpublished) data has indicated that patients with NDE have structural changes in the thalamus. We will use DKI approaches to examine thalamic and thalamocortical connectivity. Mean DKI values will be obtained from spatially co-registered regions-of-interest (principally thalamocortical regions) in standard space. We will also apply diffusion[43] and resting-state functional MRI[44] independent component analysis techniques using FSL's MELODIC toolbox[45] (http://fsl.fmrib.ox.ac.uk/fsl/fslwiki/MELODIC) and in-house MATLAB scripts to identify abnormal structural and functional thalamocortical connectivity in patients relative to controls. We will also compare patient neuropsychological and treatment outcome groups using these approaches.

White matter tracts. Our recent publications have indicated that analysis of white matter tract diffusion has significance for predicting postsurgical seizure outcome in patients with chronic focal epilepsy[12,16] and that DKI is more sensitive to tract pathology than diffusion tensor imaging in epilepsy.[46] As white matter tracts constitute the structural connections within brain networks, we will determine DKI properties along the length of multi-lobar white matter tract bundles, using our recently reported methods.[46,47]

Large scale functional networks. Using our recently described resting-state analysis techniques,[23] we will identify and analyse features of the major resting-state networks, including the fronto-parietal attentional network, default mode network, salience network, and language network. All analyses will be performed using the Functional Connectivity Toolbox.[48]

Graph theory (Connectome). The development of whole brain connectomes [49] from diffusion MRI data have led to successful data-driven approaches to predict surgical responsiveness in patients with refractory focal epilepsy from members of our

For peer review only - http://bmjopen.bmj.com/site/about/guidelines.xhtml

group.[11,13-15] Connectome approaches also support the association between postoperative seizure control and thalamocortical connectivity.[14] Similar methods have been applied to resting-state functional MRI data to model functional connectome alterations in chronic focal epilepsy.[50] As per our recent connectomic studies, whole brain structural connectomes will be generated for each participant using T1-weighted and DKI data. T1-weighted data will be parcellated into multiple regions of interest (ROI; or nodes) using Freesurfer software (http://freesurfer.net). Structural connectivity between nodes will be determined using FSL's diffusion toolbox (http://fsl.fmrib.ox.ac.uk/fsl/fslwiki/FDT) for probabilistic fibre tracking applied to diffusion MRI. Structural connectomes will be generated using the Connectome Mapping Toolkit (http://www.connectome.ch). We will use graph theory to determine global and regional network configuration. Global network "small worldness" will be assessed, representing the ratio between average nodal clustering coefficients and as network efficiency. Regional clustering coefficient, efficiency and centrality will also be calculated for key brain areas associated with seizure onset and propagation, such as thalamocortical and limbic networks. We will generate resting-state functional connectomes using a similar approach to structural connectomes. Whereas for structural connectomes edges are represented by diffusion streamlines and kurtosis diffusion scalar metrics, functional connectomes will use fMRI time series correlations between each anatomical ROI to generate a temporal correlation matrix.

Other network approaches. Using DKI, T1, and resting-state fMRI data, we will apply network-based statistics to explore network alterations common to subgroups of patients,[51] and machine/deep learning algorithms to classify individual patient outcomes.[52]

Verbal memory fMRI. Following previous work,[42] we will explore the relationship between memory deficits and neural processing of verbal memory using task-based fMRI. An event-related analysis (FSL-FEAT) will be used to examine the neural correlates of successful subsequent memory formation, comparing memory encoding networks between patients and controls, as well as between seizure-free and drug-resistant epilepsy patients. Between-group ROI comparisons in temporal and extra-temporal regions will also be made with neuropsychological variables included as covariates into a General Linear Model. We hypothesise that altered neural processing of verbal memory will be observed in patients with memory-related deficits and that network organisation differences centred around temporal regions will be observed in patients with drug-resistant epilepsy.

EEG analysis

Resting-state EEG activity will be identified by a trained clinical EEG technician. Nodes in EEG networks will be defined as electrodes, and a range of measures of interdependence between electrodes will be explored. We will apply computer models of network dynamics to resting-state EEG data.[10] In order to address Objective 1 and Objective 2, we will analyse EEG network dynamics by mirroring the approach we will take with MRI (thalamocortical and connectome analysis); firstly, we will focus on thalamocortical physiological alterations by source-localising activity within thalamic and cortical regions and determine connectivity between regions using dynamic causal modelling.[53] Secondly, we will reconstruct resting-state EEG connectomes consistent with the resting-state functional MRI approach and determine network-based physiological differences between groups. We will also explore the inter-relation between EEG and MRI determined connectivity by performing DKI tractography seeded from nodes identified as aberrantly connected using EEG to determine whether abnormal physiological connectivity is related to abnormal structural connectivity. This approach has been adopted in patients with refractory epilepsy who underwent stereoelectroencephalography.[54]

Blood and saliva sample analysis

In the laboratory, blood samples will be centrifuged within 15 minutes of collection or stored overnight at 4°c for centrifuge the following day. 250 µl aliquots will then be transferred to appropriate tubes and stored at approximately -80 degrees Celsius prior to bioanalysis. Saliva samples will be collected into an Eppendorf tube by squeezing the saturated swab using a syringe. The sample will be stored at -80 C freezer until assay. Blood and saliva samples will be analysed for inflammatory markers, HMGB1and brain-specific markers including microRNA. Inflammatory marker and HMGB1 expression analysis will be undertaken by ELISA and HMGB1 quantification will be made by liquid chromatography and mass spectrometry. Blood/saliva samples will be stored in the Liverpool University Biobank (LUB) freezer room, which is housed in the Research Technology building with LiMRIC.

For peer review only

For peer review only - http://bmjopen.bmj.com/site/about/guidelines.xhtml

Statistical analysis

We will explore the discriminatory effect of imaging biomarkers when taking into account their correlation structure and develop predictive models. (i) Cognitive dysfunction. Multivariate discriminant techniques will be used to identify structural/physiological brain measures that significantly differ between controls, patients with NDE who are cognitively normal and patients with NDE who are cognitively impaired (where impairment is defined by patient performance lower than two standard deviations [55] than that of the group of controls on respective neuropsychological tasks). We will also investigate the relationship between patient cognitive performance and imaging measures of architecture and network connectivity using multivariate regression analyses. (ii) Treatment outcome. Multivariate data techniques will be used to determine imaging measures that significantly differ between controls, patients who achieve remission over 12 months within two years, and patients who do not achieve remission within the same time period. Embedded multivariate techniques, such as least absolute shrinkage and selection operator (LASSO) and the support vector machines approaches (e.g. with radial and polynomial basis function kernels) will be applied to identify the panel of biomarkers with optimal discriminatory ability. For this process we will also consider patient demographic and clinical data, and presence of brain lesion. The variable selection algorithms will take into account the correlation between the variables, as well as variance differences across groups. To minimize the effect of possible over-fitting, penalty terms will be embedded in the variable selection algorithm to take into account model complexity. We will develop prognostic models for the assessment of outcome at two years using multivariate discriminant analysis.[56] Cross-validation and bootstrap techniques will be applied when appropriate.

Table 1 Summary of procedures

Procedure	Location	Duration	Number of examinations
1. Consent	Quiet assessment room, UoL	10 minutes	1
2. Neuropsychological evaluation	Quiet assessment room, UoL	2 hours, including comfort breaks	1
3. MRI	LiMRIC, UoL	1 hour, including safety examination and set up	1
4. Blood/saliva extraction	UoL	5 minutes	1
5. EEG	Neurophysiology, WCFT & SRFT	1 hour, including set up	1
6. Telephone questionnaire	Home	5 minutes	4 (6, 12, 18 and 24 months after scans)

ETHICS AND DISSEMINATION

Ethical approval

This study is approved by the North West, Liverpool East Research Ethics Committee (19/NW/0384) through the Integrated Research Application System (Project ID 260623). HRA approval was provided on 22/08/2019. The project is sponsored by the UoL (UoL001449) and funded by a UK Medical Research Council (MRC) research grant (MR/S00355X/1). The PI will ensure that the study is conducted in full accordance with approved protocols and that agreed modifications are disseminated to all relevant parties.

Confidentiality

Procedures for handling, processing, storage and destruction of your data are compliant with the Data Protection Act 1998. All EEG and MRI data will be anonymised prior to being exported from the WCFT, SRFT and LiMRIC, respectively. Personal information will not be identifiable from the imaging data. Names will be replaced with study ID numbers (EPINET001, EPINET002 etc), which can be backtracked to participant details using a key located at LiMRIC and only accessible to the primary care team. Storage eppendorfs will be labelled with the unique identifier only with no other patient information.

For peer review only

Similarly, all electronic neuropsychological data will be associated with study ID numbers. All documents associated with the study will be stored securely and only accessible by research staff and authorised personnel. Participant recruitment data will be monitored through study adoption by the NIHR portfolio.

Informed consent

All participants will be provided with a research information pack describing the nature and goals of the research, and study consent form, which must be completed, signed and dated. We will not recruit participants who lack capacity to provide informed consent (e.g. those with intellectual disability or dementia). Study consent forms will be retained and filed in a locked cupboard in the office of the PI. Information packs and consent forms will be given to people with epilepsy by a research nurse immediately after diagnosis in outpatient clinics. Information packs will be sent to healthy control volunteers via email or post. All participants will be given consent forms to complete at their first scanning appointment bring consent forms with them to their appointment for MRI and EEG scanning. The RT will the take the participant through the information sheet and consent form, explaining any aspects of the study that the participant is unclear about. Patients and controls will have as long as they require to consider their decision to volunteer for the research or not. The investigators contact details will be provided in the information pack.

Potential benefits and risks

There are no direct benefits to the participant. However, participation may lead to improved understanding of the aetiology, development and prognosis of epilepsy in the future.

MRI is considered a safe technique and scanning environment. The MRI system produces a high magnetic field, and it is necessary for the participants to remove all ferrometallic objects from their person before entering the scanning room. As per routine protocol, clinical members of staff screen participants for their suitability for scanning and fully debrief them after scanning. The MRI scanner is a noisy and confined environment, which may cause the participant to feel slight discomfort and claustrophobia. During scanning the subject will be monitored from the MRI control room by clinical staff and their heart-rate continuously monitored. If participants feel discomfort, scanning can be discontinued by pressing a distress button that the participant will be given before entering the scanner.

As indicated in the study information pack, scans will be reviewed to make sure that there is no brain pathology. Any MRI incidental finding will be reported to a consultant neuroradiologist. If the MRI finding is deemed to warrant further investigation, the participant's GP may be contacted. The percentage of unanticipated clinically serious brain abnormalities in healthy people is extremely low.

Venipuncture poses minimal risk of bruising or bleeding. All efforts will be made to minimise the risk of infection, appropriate training in infection control will be undertaken by all health care professionals.

There are no potential risks of EEG recordings. Neuropsychological evaluation could potentially lead to participant fatigue, frustration, or emotional disturbance. To obviate this, we will provide each participant with sufficient resting time between each assessment.

Participant engagement

All participants will be fully debriefed regarding the goals and design of the research and will have the option of receiving a letter with a brief summary of the results at the end of the study.

Dissemination

We aim to produce high-impact peer-reviewed publications of the results of the study and present findings at national and international conferences, with exclusive access to the final study dataset for a period of six years. We will target epilepsy (e.g. European Congress for Epileptology, International Epilepsy Congress, International League Against Epilepsy (ILAE) UK Chapter, American Epilepsy Society) and neuroimaging (e.g. The Organisation of Human Brain Mapping, International Society for Magnetic Resonance in Medicine) conferences. The investigators will be involved in preparing manuscripts drafts, abstracts, press releases among any other publications arising from the study and will acknowledge that the study was funded by the MRC. For each publication, only members of the research team who made a significant intellectual contribution to each piece of work

For peer review only - http://bmjopen.bmj.com/site/about/guidelines.xhtml

will be considered as an author. This is in line with journal protocol. All authors share responsibility for the contents of the submitted manuscript.

ACKNOWLEDGEMENTS

The authors are thankful to members of staff in the Institution of Translational Medicine (ITM), UoL for internal peer review of the original grant proposal and study costings for the MRC grant application. The authors acknowledge all investigators at the planned recruitment sites (WCFT and SRFT) and Dr Kumar Das (Department of Neuroradiology, WCFT), Dr Shubhabrata Biswas (Department of Neuroradiology, WCFT), Dr Surjit Lyons-Nandra (Department of Neurophysiology, WCFT), Mrs Carole Hewitt (Neuroscience Research Centre, WCFT) and Ms Nicola Leek (ITM, UoL) for their support with the study.

FOOTNOTES

Author contributions: CdB contributed to the design of the MRI procedures and prepared the protocol for publication. MGF contributed to the statistical elements of the study and sample size calculations. GB, PM and NL helped to design the neuropsychological protocol. TM and RM contributed to the overall design of the study, leading on the setup of data collection methods at recruitment sites. LB and MR contributed to the analysis plan. SK conceived the study and led the development of the protocol. All authors provided critical intellectual input to the manuscript and have approved the final version for publication.

Funding: This work is supported by the MRC grant number MR/S00355X/1 and sponsored by the UoL (4720).

Disclaimer: The trial sponsor and funders had no role in trial design; collection, management, analysis and interpretation of data; writing of the report; and the decision to submit the report for publication, nor will they have ultimate authority over any of these activities. The views and opinions expressed herein are those of the authors and do not necessarily reflect those of the UoL, MRC, and NHS.

Technical appendix: Data will be made open access after being anonymised in accordance with the MRC's open access policy.

Competing interests: None declared.

Word Count: 5263 words.

REFERENCES

1 Epilepsy E. Action. *Epilepsy facts and terminology*. https://www.epilepsy.org.uk/press/facts (accessed 10 June 2019)
2 Baker GA. The psychosocial burden of epilepsy. *Epilepsia*. 2002;43:26-30.
3 Baker GA. Depression and suicide in adolescents with epilepsy. *Neurology*. 2006;66(66 suppl 3):S5-12.
4 Surges R, Sander JW. Sudden unexpected death in epilepsy: mechanisms, prevalence, and prevention. *Current opinion in neurology*. 2012;25(2):201-7.
5 Meador KJ, French J, Loring DW, Pennell PB. Disparities in NIH funding for epilepsy research. *Neurology*. 2011;77(13):1305-7.
6 Pohlmann-Eden B, Crocker CE, Schmidt MH. A conceptual framework for the use of neuroimaging to study and predict pharmacoresistance in epilepsy. *Epilepsia*. 2013;54:75-9.
7 Richardson MP. Large scale brain models of epilepsy: dynamics meets connectomics. *J Neurol Neurosurg Psychiatry*. 2012;83(12):1238-48.
8 Bernhardt BC, Bonilha L, Gross DW. Network analysis for a network disorder: the emerging role of graph theory in the study of epilepsy. *Epilepsy & Behavior*. 2015;50:162-70.
9 Terry JR, Benjamin O, Richardson MP. Seizure generation: the role of nodes and networks. *Epilepsia*. 2012;53(9):e166
10 Schmidt H, Woldman W, Goodfellow M, Chowdhury FA, Koutroumanidis M, Jewell S, Richardson MP, Terry JR. A computational biomarker of idiopathic generalized epilepsy from resting state EEG. *Epilepsia*. 2016;57(10):e200-4.

For peer review only - http://bmjopen.bmj.com/site/about/guidelines.xhtml

11Hutchings F, Han CE, Keller SS, Weber B, Taylor PN, Kaiser M. Predicting surgery targets in temporal lobe epilepsy through structural connectome based simulations. *PLoS computational biology*. 2015;11(12):e1004642.

12Keller SS, Richardson MP, Schoene-Bake JC, O'muircheartaigh J, Elkommos S, Kreilkamp B, Goh YY, Marson AG, Elger C, Weber B. Thalamotemporal alteration and postoperative seizures in temporal lobe epilepsy. *Annals of neurology*. 2015;77(5):760-74.

13Munsell BC, Wee CY, Keller SS, Weber B, Elger C, da Silva LA, Nesland T, Styner M, Shen D, Bonilha L. Evaluation of machine learning algorithms for treatment outcome prediction in patients with epilepsy based on structural connectome data. *Neuroimage*. 2015;118:219-30.

14Bonilha L, Helpern JA, Sainju R, Nesland T, Edwards JC, Glazier SS, Tabesh A. Presurgical connectome and postsurgical seizure control in temporal lobe epilepsy. *Neurology*. 2013;81(19):1704-10.

15Bonilha L, Jensen JH, Baker N, Breedlove J, Nesland T, Lin JJ, Drane DL, Saindane AM, Binder JR, Kuzniecky RI. The brain connectome as a personalized biomarker of seizure outcomes after temporal lobectomy. *Neurology*. 2015;84(18):1846-53.

16Keller SS, Glenn GR, Weber B, Kreilkamp BA, Jensen JH, Helpern JA, Wagner J, Barker GJ, Richardson MP, Bonilha L. Preoperative automated fibre quantification predicts postoperative seizure outcome in temporal lobe epilepsy. *Brain*. 2016;140(1):68-82.

17Maroso M, Balosso S, Ravizza T, et al. Toll-like receptor 4 and high-mobility group box-1 are involved in ictogenesis and can be targeted to reduce seizures. *Nature medicine*. 2010;14(4):413.

18Balosso S. Disulfide-containing high mobility group box-1 promotes N-methyl-D-aspartate receptor function and excitotoxicity by activating Toll-like receptor 4-dependent signaling in hippocampal neurons. Antioxid Redox Signal; 2014.

19Kelley MS, Jacobs MP, Lowenstein DH, NINDS Epilepsy Benchmark Stewards. The NINDS epilepsy research benchmarks. *Epilepsia*. 2009;50(3):579-82.

20Long C, Fureman B, Dingledine R. 2014 Epilepsy benchmarks: progress and opportunities. *Epilepsy currents*. 2016;16(3):179-81.

21Brodie, M.J., Road to refractory epilepsy: the Glasgow story. *Epilepsia*, 2013;54 Suppl 2:5-8.

22Mohanraj, R. and M.J. Brodie, Early predictors of outcome in newly diagnosed epilepsy. *Seizure*, 2013;22(5):333-44.

23Alonazi BK, Keller SS, Fallon N, Adams V, Das K, Marson AG, Sluming V. Resting-state functional brain networks in adults with a new diagnosis of focal epilepsy. *Brain and behavior*. 2019;9(1):e01168.

24Machin DC, Tan SB, Tan SH. *Sample size Tables for Clinical studies.* Wiley-Blackwell; 2009.

25Van Paesschen W, Duncan JS, Stevens JM, Connelly A. Etiology and early prognosis of newly diagnosed partial seizures in adults: a quantitative hippocampal MRI study. *Neurology*. 1997;49(3):753-7.

26Van Paesschen W, Duncan JS, Stevens JM, Connelly A. Longitudinal quantitative hippocampal magnetic resonance imaging study of adults with newly diagnosed partial seizures: one-year follow-up results. *Epilepsia*. 1998;39(6):633-9.

27Jacoby A, Lane S, Marson A, Baker GA, MESS Study Group. Relationship of clinical and quality of life trajectories following the onset of seizures: findings from the UK MESS Study. *Epilepsia*. 2011;52(5):965-74.

28Marson AG, Al-Kharusi AM, Alwaidh M, Appleton R, Baker GA, Chadwick DW, Cramp C, Cockerell OC, Cooper PN, Doughty J, Eaton B. The SANAD study of effectiveness of valproate, lamotrigine, or topiramate for generalised and unclassifiable epilepsy: an unblinded randomised controlled trial. *The Lancet*. 2007;369(9566):1016-26.

29SANADII [Internet]. Standard and New Antiepileptic Drugs Trial II Website [cited 2019 Jul 17]. Available from: http://www.sanad2.org.uk

30Taylor J, Kolamunnage-Dona R, Marson AG, Smith PE, Aldenkamp AP, Baker GA, SANAD Study Group. Patients with epilepsy: cognitively compromised before the start of antiepileptic drug treatment?. *Epilepsia*. 2010;51(1):48-56.

31Eriksson SH, Thom M, Symms MR, Focke NK, Martinian L, Sisodiya SM, Duncan JS. Cortical neuronal loss and hippocampal sclerosis are not detected by voxel-based morphometry in individual epilepsy surgery patients. *Human brain mapping*. 2009;30(10):3351-60.

32Mohanraj R, Brodie MJ. Early predictors of outcome in newly diagnosed epilepsy. *Seizure*. 2013;22(5):333-44.

For peer review only

33 Bonnett LJ, Powell GA, Smith CT, Marson AG. Risk of a seizure recurrence after a breakthrough seizure and the implications for driving: further analysis of the standard versus new antiepileptic drugs (SANAD) randomised controlled trial. *BMJ open*. 2017;7(7):e015868.

34 Baker GA, Taylor J, Aldenkamp AP. Newly diagnosed epilepsy: cognitive outcome after 12 months. *Epilepsia*. 2011;52(6):1084-91.

35 Drozdick LW, Holdnack JA, Weiss LG, Zhou X. Overview of the WAIS–IV/WMS–IV/ACS. *InWAIS-IV, WMS-IV, and ACS.* 2013; (pp. 1-73). Academic Press.

36 Swanson J. The Delis-Kaplan executive function system: a review. *Canadian Journal of School Psychology*. 2005;20(1-2):117-28.

37 Scarpina F, Tagini S. *The stroop color and word test. Frontiers in psychology*. 2017;8:557.

38 Fiest KM, Patten SB, Wiebe S, Bulloch AG, Maxwell CJ, Jetté N. Validating screening tools for depression in epilepsy. *Epilepsia*. 2014;55(10):1642-50.

39 Seo JG, Cho YW, Lee SJ, Lee JJ, Kim JE, Moon HJ, Park SP. Validation of the generalized anxiety disorder-7 in people with epilepsy: a MEPSY study. *Epilepsy & Behavior*. 2014;35:59-63.

40 Brooks J, Baker GA, Aldenkamp AP. The A-B Neuropsychological Assessment Schedule (ABNAS): the further refinement of a patient-based scale of patient-perceived cognitive functioning. *Epilepsy Research.* 2001;43:227-237.

41 Ridsdale L, Wojewodka G, Robinson E, Landau S, Noble A, Taylor S, Richardson M, Baker G, Goldstein LH, SMILE Team. Characteristics associated with quality of life among people with drug-resistant epilepsy. Journal of neurology. 2017;264(6):1174-84.93

42 Sidhu MK, Stretton J, Winston GP, Bonelli S, Centeno M, Vollmar C, Symms M, Thompson PJ, Koepp MJ, Duncan JS. A functional magnetic resonance imaging study mapping the episodic memory encoding network in temporal lobe epilepsy. *Brain*. 2013;136(6):1868-88.

43 O'Muircheartaigh J, Vollmar C, Traynor C, Barker GJ, Kumari V, Symms MR, Thompson P, Duncan JS, Koepp MJ, Richardson MP. Clustering probabilistic tractograms using independent component analysis applied to the thalamus. *Neuroimage*. 2011;54(3):2020-32

44 O'Muircheartaigh J, Keller SS, Barker GJ, Richardson MP. White matter connectivity of the thalamus delineates the functional architecture of competing thalamocortical systems. *Cerebral Cortex*. 2015;25(11):4477-89.

45 Beckmann CF, Smith SM. Tensorial extensions of independent component analysis for multisubject FMRI analysis. *Neuroimage*. 2005;25(1):294-311.

46 Glenn GR, Jensen JH, Helpern JA, Spampinato MV, Kuzniecky R, Keller SS, Bonilha L. Epilepsy-related cytoarchitectonic abnormalities along white matter pathways. *J Neurol Neurosurg Psychiatry*. 2016;87(9):930-6.

47 Kreilkamp BA, Weber B, Richardson MP, Keller SS. Automated tractography in patients with temporal lobe epilepsy using TRActs Constrained by UnderLying Anatomy (TRACULA). *NeuroImage*: Clinical. 2017;14:67-76.

48 Whitfield-Gabrieli S, Nieto-Castanon A. Conn: a functional connectivity toolbox for correlated and anticorrelated brain networks. *Brain connectivity*. 2012;2(3):125-41.

49 Sporns O. *Discovering the human connectome*. MIT press; 2012.

50 Liao W, Ji GJ, Xu Q, Wei W, Wang J, Wang Z, Yang F, Sun K, Jiao Q, Richardson MP, Zang YF. Functional connectome before and following temporal lobectomy in mesial temporal lobe epilepsy. *Scientific reports*. 2016;6:23153.

51 Zalesky A, Fornito A, Bullmore ET. Network-based statistic: identifying differences in brain networks. *Neuroimage*. 2010;53(4):1197-207.

52 Munsell BC, Wee CY, Keller SS, Weber B, Elger C, da Silva LA, Nesland T, Styner M, Shen D, Bonilha L. Evaluation of machine learning algorithms for treatment outcome prediction in patients with epilepsy based on structural connectome data. *Neuroimage*. 2015;118:219-30.

53 Kiebel SJ, Garrido MI, Moran RJ, Friston KJ. Dynamic causal modelling for EEG and MEG. *Cognitive neurodynamics*. 2008;2(2):121.

54 Besson P, Bandt SK, Proix T, Lagarde S, Jirsa VK, Ranjeva JP, Bartolomei F, Guye M. Anatomic consistencies across epilepsies: a stereotactic-EEG informed high-resolution structural connectivity study. *Brain*. 2017;140(10):2639-52.

For peer review only

55Goldman JG, Holden S, Bernard B, Ouyang B, Goetz CG, Stebbins GT. Defining optimal cutoff scores for cognitive impairment using Movement Disorder Society Task Force criteria for mild cognitive impairment in Parkinson's disease. *Movement Disorders*. 2013;28(14):1972-9.

56Hughes DM, Komárek A, Bonnett LJ, Czanner G, García-Fiñana M. Dynamic classification using credible intervals in longitudinal discriminant analysis. *Statistics in medicine*. 2017;36(24):3858-74.

LIST OF FIGURES

1 The recruitment process 16
2 Organisation of the study phases 17

For peer review only

For peer review only - http://bmjopen.bmj.com/site/about/guidelines.xhtml

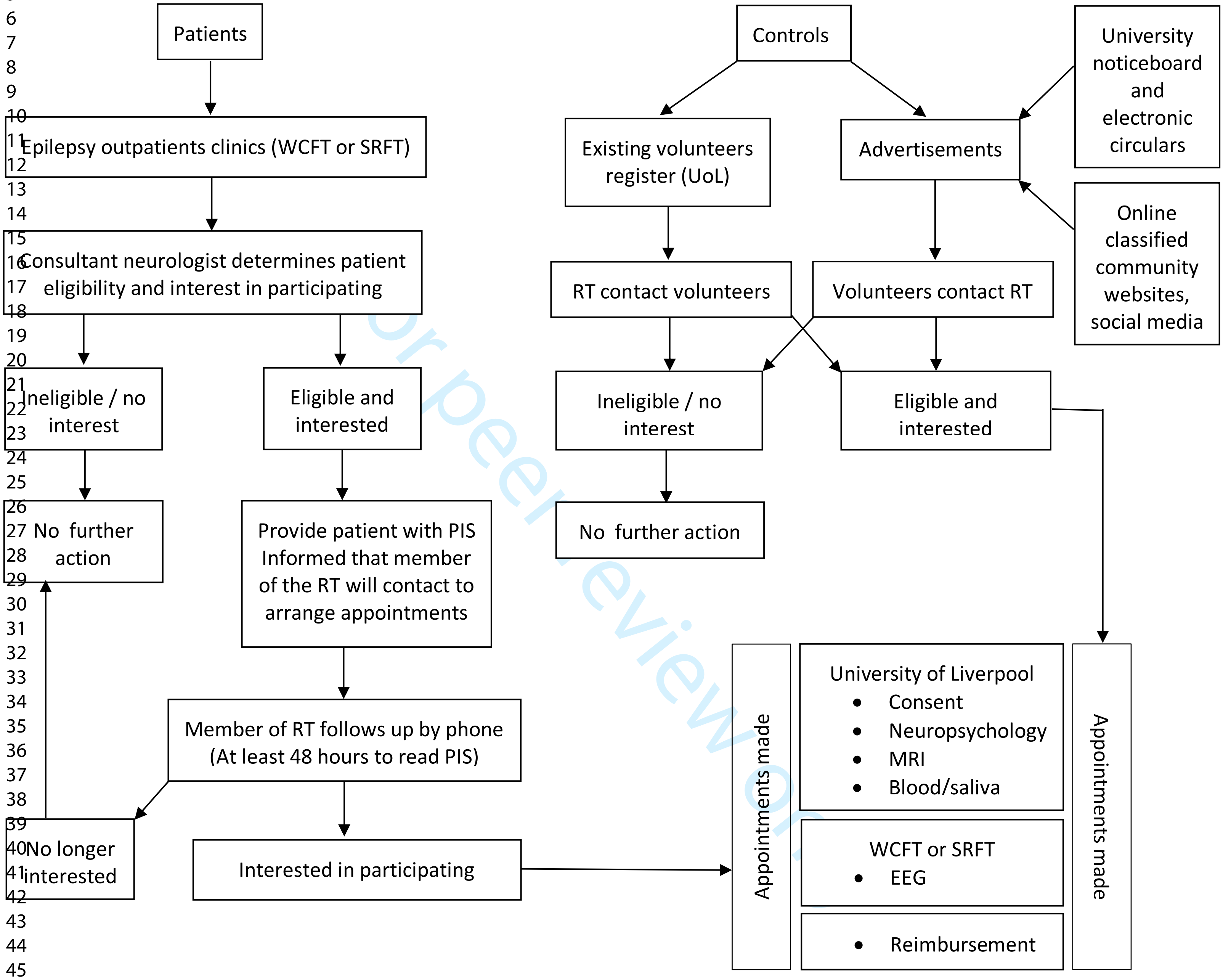
Patients
Epilepsy outpatients clinics (WCFT or SRFT)
Consultant neurologist determines patient eligibility and interest in participating
Ineligible / no interest
No further action
Eligible and interested
Provide patient with PIS Informed that member of the RT will contact to arrange appointments
Member of RT follows up by phone (At least 48 hours to read PIS)
No longer interested
Interested in participating
Controls
Existing volunteers register (UoL)
Advertisements
University noticeboard and electronic circulars
Online classified community websites, social media
RT contact volunteers
Volunteers contact RT
Ineligible / no interest
Eligible and interested
No further action
Appointments made
University of Liverpool
Consent
Neuropsychology
MRI
Blood/saliva
WCFT or SRFT
EEG
Reimbursement
Appointments made

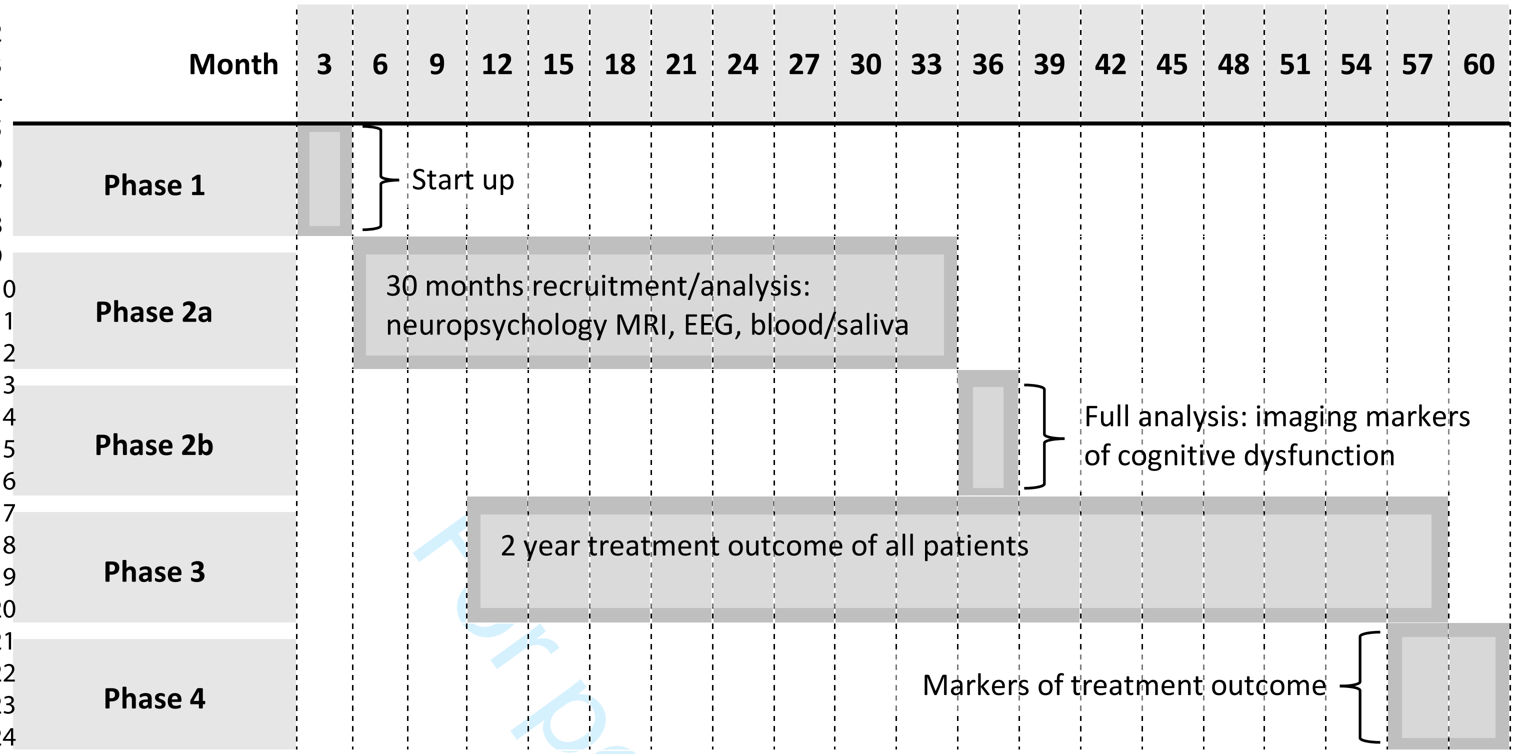

For peer review only

Reporting checklist for protocol of a clinical trial.

Based on the SPIRIT guidelines.

Instructions to authors

Complete this checklist by entering the page numbers from your manuscript where readers will find each of the items listed below.

Your article may not currently address all the items on the checklist. Please modify your text to include the missing information. If you are certain that an item does not apply, please write "n/a" and provide a short explanation.

Upload your completed checklist as an extra file when you submit to a journal.

In your methods section, say that you used the SPIRITreporting guidelines, and cite them as:

Chan A-W, Tetzlaff JM, Altman DG, Laupacis A, Gøtzsche PC, Krleža-Jerić K, Hróbjartsson A, Mann H, Dickersin K, Berlin J, Doré C, Parulekar W, Summerskill W, Groves T, Schulz K, Sox H, Rockhold FW, Rennie D, Moher D. SPIRIT 2013 Statement: Defining standard protocol items for clinical trials. Ann Intern Med. 2013;158(3):200-207

		Reporting Item	Page Number
**Administrative information**			
Title	#1	Descriptive title identifying the study design, population, interventions, and, if applicable, trial acronym	2
Trial registration	#2a	Trial identifier and registry name. If not yet registered, name of intended registry	2
Trial registration: data set	#2b	All items from the World Health Organization Trial Registration Data Set	n/a
Protocol version	#3	Date and version identifier	2
Funding	#4	Sources and types of financial, material, and other support	2
Roles and responsibilities: contributorship	#5a	Names, affiliations, and roles of protocol contributors	12

For peer review only

Roles and responsibilities: sponsor contact information	#5b	Name and contact information for the trial sponsor	2
Roles and responsibilities: sponsor and funder	#5c	Role of study sponsor and funders, if any, in study design; collection, management, analysis, and interpretation of data; writing of the report; and the decision to submit the report for publication, including whether they will have ultimate authority over any of these activities	12
Roles and responsibilities: committees	#5d	Composition, roles, and responsibilities of the coordinating centre, steering committee, endpoint adjudication committee, data management team, and other individuals or groups overseeing the trial, if applicable (see Item 21a for data monitoring committee)	12
**Introduction**			
Background and rationale	#6a	Description of research question and justification for undertaking the trial, including summary of relevant studies (published and unpublished) examining benefits and harms for each intervention	2-3
Background and rationale: choice of comparators	#6b	Explanation for choice of comparators	3
Objectives	#7	Specific objectives or hypotheses	3
Trial design	#8	Description of trial design including type of trial (eg, parallel group, crossover, factorial, single group), allocation ratio, and framework (eg, superiority, equivalence, non-inferiority, exploratory)	3
**Methods: Participants, interventions, and outcomes**			
Study setting	#9	Description of study settings (eg, community clinic, academic hospital) and list of countries where data will be	4

For peer review only

		collected. Reference to where list of study sites can be obtained	
Eligibility criteria	#10	Inclusion and exclusion criteria for participants. If applicable, eligibility criteria for study centres and individuals who will perform the interventions (eg, surgeons, psychotherapists)	4
Interventions: description	#11a	Interventions for each group with sufficient detail to allow replication, including how and when they will be administered	6-8
Interventions: modifications	#11b	Criteria for discontinuing or modifying allocated interventions for a given trial participant (eg, drug dose change in response to harms, participant request, or improving / worsening disease)	n/a Observational cohort study
Interventions: adherance	#11c	Strategies to improve adherence to intervention protocols, and any procedures for monitoring adherence (eg, drug tablet return; laboratory tests)	n/a Observational cohort study
Interventions: concomitant care	#11d	Relevant concomitant care and interventions that are permitted or prohibited during the trial	n/a<br><br>Observational cohort study
Outcomes	#12	Primary, secondary, and other outcomes, including the specific measurement variable (eg, systolic blood pressure), analysis metric (eg, change from baseline, final value, time to event), method of aggregation (eg, median, proportion), and time point for each outcome. Explanation of the clinical relevance of chosen efficacy and harm outcomes is strongly recommended	6
Participant timeline	#13	Time schedule of enrolment, interventions (including any run-ins and washouts), assessments, and visits for participants. A schematic diagram is highly recommended (see Figure)	6
Sample size	#14	Estimated number of participants needed to achieve study objectives and how it was determined, including clinical and statistical assumptions supporting any sample size calculations	5

For peer review only

Recruitment	#15	Strategies for achieving adequate participant enrolment to reach target sample size	5
**Methods: Assignment of interventions (for controlled trials)**			
Allocation: sequence generation	#16a	Method of generating the allocation sequence (eg, computer-generated random numbers), and list of any factors for stratification. To reduce predictability of a random sequence, details of any planned restriction (eg, blocking) should be provided in a separate document that is unavailable to those who enrol participants or assign interventions	n/a<br><br>Observational cohort study
Allocation concealment mechanism	#16b	Mechanism of implementing the allocation sequence (eg, central telephone; sequentially numbered, opaque, sealed envelopes), describing any steps to conceal the sequence until interventions are assigned	n/a<br><br>Observational cohort study
Allocation: implementation	#16c	Who will generate the allocation sequence, who will enrol participants, and who will assign participants to interventions	n/a<br><br>Observational cohort study
Blinding (masking)	#17a	Who will be blinded after assignment to interventions (eg, trial participants, care providers, outcome assessors, data analysts), and how	n/a<br><br>Observational cohort study
Blinding (masking): emergency unblinding	#17b	If blinded, circumstances under which unblinding is permissible, and procedure for revealing a participant's allocated intervention during the trial	n/a<br><br>Observational cohort study
**Methods: Data collection, management, and analysis**			
Data collection plan	#18a	Plans for assessment and collection of outcome, baseline, and other trial data, including any related processes to promote data quality (eg, duplicate measurements, training	5-8

For peer review only

		of assessors) and a description of study instruments (eg, questionnaires, laboratory tests) along with their reliability and validity, if known. Reference to where data collection forms can be found, if not in the protocol	
Data collection plan: retention	#18b	Plans to promote participant retention and complete follow-up, including list of any outcome data to be collected for participants who discontinue or deviate from intervention protocols	5
Data management	#19	Plans for data entry, coding, security, and storage, including any related processes to promote data quality (eg, double data entry; range checks for data values). Reference to where details of data management procedures can be found, if not in the protocol	10
Statistics: outcomes	#20a	Statistical methods for analysing primary and secondary outcomes. Reference to where other details of the statistical analysis plan can be found, if not in the protocol	8-10
Statistics: additional analyses	#20b	Methods for any additional analyses (eg, subgroup and adjusted analyses)	8-10
Statistics: analysis population and missing data	#20c	Definition of analysis population relating to protocol non-adherence (eg, as randomised analysis), and any statistical methods to handle missing data (eg, multiple imputation)	8-10
**Methods: Monitoring**			
Data monitoring: formal committee	#21a	Composition of data monitoring committee (DMC); summary of its role and reporting structure; statement of whether it is independent from the sponsor and competing interests; and reference to where further details about its charter can be found, if not in the protocol. Alternatively, an explanation of why a DMC is not needed	11
Data monitoring: interim analysis	#21b	Description of any interim analyses and stopping guidelines, including who will have access to these interim results and make the final decision to terminate the trial	n/a<br><br>No interim analysis planned

For peer review only

Harms	#22	Plans for collecting, assessing, reporting, and managing solicited and spontaneously reported adverse events and other unintended effects of trial interventions or trial conduct	11
Auditing	#23	Frequency and procedures for auditing trial conduct, if any, and whether the process will be independent from investigators and the sponsor	n/a<br>No auditing trial conduct
**Ethics and dissemination**			
Research ethics approval	#24	Plans for seeking research ethics committee / institutional review board (REC / IRB) approval	10
Protocol amendments	#25	Plans for communicating important protocol modifications (eg, changes to eligibility criteria, outcomes, analyses) to relevant parties (eg, investigators, REC / IRBs, trial participants, trial registries, journals, regulators)	10
Consent or assent	#26a	Who will obtain informed consent or assent from potential trial participants or authorised surrogates, and how (see Item 32)	10
Consent or assent: ancillary studies	#26b	Additional consent provisions for collection and use of participant data and biological specimens in ancillary studies, if applicable	n/a
Confidentiality	#27	How personal information about potential and enrolled participants will be collected, shared, and maintained in order to protect confidentiality before, during, and after the trial	10-11
Declaration of interests	#28	Financial and other competing interests for principal investigators for the overall trial and each study site	12
Data access	#29	Statement of who will have access to the final trial dataset, and disclosure of contractual agreements that limit such access for investigators	11
Ancillary and post trial care	#30	Provisions, if any, for ancillary and post-trial care, and for compensation to those who suffer harm from trial participation	n/a

For peer review only

			Observational cohort study
Dissemination policy: trial results	#31a	Plans for investigators and sponsor to communicate trial results to participants, healthcare professionals, the public, and other relevant groups (eg, via publication, reporting in results databases, or other data sharing arrangements), including any publication restrictions	11
Dissemination policy: authorship	#31b	Authorship eligibility guidelines and any intended use of professional writers	11
Dissemination policy: reproducible research	#31c	Plans, if any, for granting public access to the full protocol, participant-level dataset, and statistical code	n/a
**Appendices**			
Informed consent materials	#32	Model consent form and other related documentation given to participants and authorised surrogates	n/a
Biological specimens	#33	Plans for collection, laboratory evaluation, and storage of biological specimens for genetic or molecular analysis in the current trial and for future use in ancillary studies, if applicable	9

None The SPIRIT checklist is distributed under the terms of the Creative Commons Attribution License CC-BY-ND 3.0. This checklist can be completed online using https://www.goodreports.org/, a tool made by the EQUATOR Network in collaboration with Penelope.ai

For peer review only

BMJ Open

A protocol for an observational cohort study in the UK investigating imaging network markers of cognitive dysfunction and pharmacoresistance in newly diagnosed epilepsy.

Journal:	*BMJ Open*
Manuscript ID	bmjopen-2019-034347.R1
Article Type:	Protocol
Date Submitted by the Author:	23-Sep-2019
Complete List of Authors:	de Bézenac, Christophe; University of Liverpool, Molecular and Clinical Pharmacology, Institute of Translational Medicine<br>Garcia-Finana, Marta; University of Liverpool, Biostatistics<br>Baker, Gus; University of Liverpool Faculty of Health and Life Sciences, Molecular and Clinical Pharmacology<br>Moore, Perry; Salford Royal NHS Foundation Trust<br>Leek, Nicola; University of Liverpool, 1. Department of Molecular and Clinical Pharmacology, Institute of Translational Medicine<br>Mohanraj, Rajiv; Salford Royal NHS Foundation Trust<br>Bonilha, Leonardo ; Medical University of South Carolina, Department of Neurology<br>Richardson, Mark; King's College London, Institute of Psychiatry, Psychology & Neuroscience<br>Marson, Anthony; University of Liverpool, Molecular and Clinical Pharmacology<br>Keller, Simon; University of Liverpool, Department of Molecular and Clinical Pharmacology, Institute of Translational Medicine
**Primary Subject Heading**:	Neurology
Secondary Subject Heading:	Pathology
Keywords:	Epilepsy < NEUROLOGY, Brain connectivity, Neuropsychology, Inflammation, Newly diagnosed

SCHOLARONE™
Manuscripts

A protocol for an observational cohort study in the UK investigating imaging network markers of cognitive dysfunction and pharmacoresistance in newly diagnosed epilepsy.

Christophe de Bézenac[1], Marta García-Fiñana[2], Gus A. Baker[1,3], Perry Moore[4], Nicola Leek[1], Rajiv Mohanraj[4], Leonardo Bonilha[5], Mark P. Richardson[6], Anthony G. Marson[1,3] and Simon S. Keller[1,3]

1. Department of Molecular and Clinical Pharmacology, Institute of Translational Medicine, University of Liverpool, Liverpool, L69 3BX, United Kingdom.
2. Department of Biostatistics, Institute of Translational Medicine, University of Liverpool, Liverpool, L69 3BX, United Kingdom.
3. The Walton Centre NHS Foundation Trust, Lower Lane, Liverpool, L9 7LJ, United Kingdom.
4. Salford Royal NHS Foundation Trust, Salford M6 8HD, United Kingdom.
5. Department of Neurology, Medical University of South Carolina, Charleston, USA
6. Department of Basic and Clinical Neuroscience, Institute of Psychiatry, Psychology and Neuroscience, King's College London, London, UK

Christophe de Bézenac: cedb@liverpool.ac.uk; +44 (0)151 529 5943 (corresponding author)
Marta García-Fiñana: martaf@liverpool.ac.uk; +44 (0)151 794 9755.
Gus Barker: gus@liverpool.ac.uk; +44 (0)151 529 5948
Perry Moore: Perry.Moore@srft.nhs.uk; +44 (0)161 789 7373
Nicola Leek: nleek@liverpool.ac.uk; +44 (0)151 529 5943
Rajiv Mohanraj: Rajiv.Mohanraj@srft.nhs.uk; +44 (0)161 206 4626
Leonardo Bonilha: bonilha@musc.edu; +1 843 876 8311
Mark Richardson: mark.richardson@kcl.ac.uk; +44 (0)207 848 5429
Anthony Marson: marjon01@liverpool.ac.uk; +44 (0)151 529 5705
Simon Keller: Simon.keller@liverpool.ac.uk; +44 (0)151 529 5943

Correspondence to
Christophe de Bézenac,
Liverpool Magnetic Resonance
Imaging Centre, University of
Liverpool, Pembroke Place,
Liverpool, L69 3GE UK
cedb@liverpool.ac.uk;
+44 (0)151 529 5943

For peer review only - http://bmjopen.bmj.com/site/about/guidelines.xhtml

ABSTRACT

Introduction: Epilepsy is one of the most common serious brain disorders, characterised by seizures that severely affect a person's quality of life and, frequently, their cognitive and mental health. Although most existing work has examined chronic epilepsy, newly diagnosed patients presents a unique opportunity to understand the underlying biology of epilepsy and predict effective treatment pathways. The objective of this prospective cohort study is to examine whether cognitive dysfunction is associated with measurable brain architectural and connectivity impairments at diagnosis and whether the outcome of antiepileptic drug treatment can be predicted using these measures.

Methods and analysis: 107 patients with newly diagnosed focal epilepsy from two NHS Trusts and 48 healthy controls (aged 16 to 65 years) will be recruited over a period of 30 months. Baseline assessments will include neuropsychological evaluation, structural and functional MRI, EEG, and a blood and saliva sample. Patients will be followed up every 6 months for a 24-month period to assess treatment outcomes. Connectivity- and network-based analyses of EEG and MRI data will be carried out and examined in relation to neuropsychological evaluation and patient treatment outcomes. Patient outcomes will also be investigated with respect to analysis of molecular isoforms of high mobility group box-1 (HMGB1) from blood and saliva samples.

Ethics and dissemination: This study was approved by the North West, Liverpool East Research Ethics Committee (19/NW/0384) and funded by a Medical Research Council research grant (MR/S00355X/1). Findings will be presented at national and international meetings and conferences and published in peer-reviewed journals.

Trial registration number: IRAS Project ID 260623; Protocol Version 6; Pre-results.

Keywords: Newly diagnosed, Epilepsy, Brain connectivity, Neuropsychology, Inflammation

ARTICLE SUMMARY

Strengths and limitations of this study

- This will be the first study to prospectively investigate brain structural and physiological architecture and connectivity in adults with a new diagnosis of focal epilepsy
- The study is expected to provide insights into the biology underlying cognitive dysfunction in the early stages of human epilepsy, and to lead to the development of prognostic markers of future pharmacoresistance.
- Expected recruitment has been based on records of past diagnosis at recruitment sites and while the study is expected to recruit well, unexpected under-recruitment is possible and would be a barrier to timely completion.
- A second potential limitation of this study is the potential for participant attrition and loss of patient follow up at multiple points over 24 months; missing data could impact on the validity of study conclusions.

INTRODUCTION

Background and rationale

Epilepsy is one of the most common serious brain disorders; every day in the UK, 87 people are diagnosed with epilepsy, affecting over 600,000 people.[1] The condition is characterised by devastating seizures that severely impact on a person's quality of life. Epilepsy frequently affects a person's cognitive and mental health,[2] and the disorder contributes to elevated propensity for depression, suicide and sudden and unexpected death compared to the general population.[3,4] Despite this, research into epilepsy has been grossly underfunded compared to other medical conditions of similar economic, social and personal impact.[5] The vast majority of existing work in human studies has been performed in chronic epilepsy. Newly diagnosed epilepsy (NDE) is only rarely studied despite representing a key point in time to understand the underlying biology of the disorder in the absence of confounds including anti-epileptic drugs (AEDs) and long term seizure effects.[6] It is important to understand the reasons why people with epilepsy experience cognitive problems and seizures after treatment using safe imaging technologies from the earliest time point of the disorder. If we can understand these reasons in the early stages of epilepsy, we may be able to predict which patients will continue to experience seizures despite standard drug therapy. Patients who will not respond to drug therapy could potentially be offered alternative or adjunctive treatments, saving time, cost, and the experience of undesirable side effects of certain AEDs.

For peer review only - http://bmjopen.bmj.com/site/about/guidelines.xhtml

MRI and EEG are routinely used to assess people with epilepsy. However, the application of these brain imaging techniques in the context of standard care cannot determine why some patients have cognitive problems and why others do not, and why some patients do not respond to AED therapy while others do. A new direction of brain imaging is therefore required; preferably one that can be incorporated into the standard clinical evaluation of patients. In patients with longstanding epilepsy the study of brain connectivity and networks (how different regions of the brain work together by virtue of their connectivity) using MRI and EEG has recently provided valuable insights into how the brain is structurally and physiologically altered in epilepsy.[7,8] There is growing evidence that aberrant network dynamics are a key part of the underlying mechanisms of focal and generalised epilepsies.[9] State-of-the-art quantitative structural (e.g. diffusion MRI and tractography approaches), functional (e.g. resting-state functional MRI), and physiological (e.g. EEG) imaging techniques have provided a novel way of automatically distinguishing longstanding epilepsy patients from healthy controls,[10] and predicting postoperative treatment outcome in severe epilepsy.[11-16] We propose that these approaches will provide new explanations for the causes of cognitive problems and future treatment outcome from the beginning of a patient's life with epilepsy.

Furthermore, mechanistic blood and saliva biomarkers could greatly enhance drug discovery by providing novel therapeutic targets and enrich trial populations, facilitating early surgical evaluation in drug-resistance. We have soon-to-be-published data suggesting that molecular isoforms of high mobility group box-1 (HMGB1) – a protein critically involved in the initiation of the inflammatory cascade in epilepsy –[17] have potential as a prognostic biomarker. The acetylated, disulfide form of HMGB1, which triggers pro-inflammatory cytokine release via toll-like receptor 4, has shown pathological effects in pre-clinical models of seizures.[18] In a parallel running study, we are currently studying people with longstanding epilepsy using MRI and blood serum markers of HMGB1 (Short title: "MRI of inflammation in epilepsy"; IRAS project ID 220138; REC reference 17/NW/0342, Northwest-Liverpool).

This observational cohort study will be the first to prospectively investigate brain structural and physiological architecture and connectivity in adults with NDE with overarching goals to: (1) understand the neural basis of cognitive impairment; and (2) identify why and in whom seizures persist despite AED therapy. We will recruit adults with a new diagnosis of focal epilepsy and perform cognitive assessment and sophisticated analysis of MRI and EEG data. At the time of scanning and neuropsychological evaluation (baseline), all participants will additionally have blood and saliva extracted. Patients will be followed up longitudinally to determine their response to AED therapy. MRI/EEG data will be used to identify the neural correlates of cognitive impairment and to predict treatment outcome. Data generated from extracted blood and saliva samples will also be used to predict treatment outcome. To remain consistent with our ongoing work that investigates the correlation between MRI data in HMGB1 in people with epilepsy, we will use an identical approach of data acquisition and analysis. This work will be performed in an environment with demonstrated excellence in the care of people with epilepsy, recruitment of adults with NDE into clinical trials, and expertise in MRI, EEG, neuropsychological and blood serum analysis. The research objectives of the proposed work directly address internationally agreed research priorities in epilepsy, with potential to provide significant insights into the epilepsy phenotype and to generate clinically meaningful non-invasive markers of treatment outcome.[19,20]

Study objectives and design

The goal of the proposed research is to perform the first prospective multi-modal imaging investigation of brain architecture and connectivity in adults with a new diagnosis of focal epilepsy. The project aims to provide new insights into the biology underlying cognitive dysfunction in the early stages of human epilepsy and develop prognostic markers of future pharmacoresistance. The research will take place in context of a collaborative research and clinical environment that has demonstrated excellence in the recruitment and study of patients with newly diagnosed epilepsy. The three main objectives are outlined below.

Objective 1

The primary objective is to determine the cognitive phenotype associated with newly diagnosed epilepsy and whether cognitive dysfunction is associated with measurable brain architectural and connectivity impairments at diagnosis. We expect that patients will be cognitively impaired in the domains of memory, sustained attention and executive function; this impairment will be reflected in pathological alterations to structural and functional neural networks and responses to a verbal memory task computed from f/MRI and EEG.

For peer review only - http://bmjopen.bmj.com/site/about/guidelines.xhtml

Objective 2

A secondary objective is to determine whether AED treatment outcome can be predicted using multi-modal imaging measures of brain architecture and connectivity at the point of epilepsy diagnosis. We expect that architectural and physiological alterations within local and networked brain regions can predict patient response to pharmacological therapy at diagnosis.

Objective 3

We will determine whether blood serum and saliva derived measures of inflammation can predict AED treatment outcome in patients with newly diagnosed epilepsy and examine relationships between molecular isoforms of HMGB1 and MRI, EEG and neuropsychological data.

METHODS AND ANALYSIS

Study environment

Research will be carried out by the Epilepsy Research Group within the Institute of Translational Medicine (ITM), University of Liverpool (UoL). The group is closely affiliated with the Walton Centre (WCFT) Foundation NHS Trust from where patients will be recruited, alongside the Salford Royal NHS Foundation Trust (SRFT). Both WCFT and SRFT will acquire patient EEG data in context of standard clinical care; EEG data for healthy controls will be acquired at the WCFT. MRI acquisition will be performed at the Liverpool Magnetic Resonance Imaging Centre (LiMRIC; www.liv.ac.uk/limric), using a Siemens Prisma 3T scanner. Blood/saliva will be extracted from participants at LiMRC and stored at the Liverpool University Biobank freezer room.

Eligibility criteria

Based on sample size calculations (see below), we will recruit 107 people with a new diagnosis of focal epilepsy and 48 healthy controls. Inclusion and exclusion criteria for patients and controls are outlined below.

Inclusion Criteria

Patients with epilepsy

- Patients who are attending or have attended clinics at WCFT and SRFT who have been diagnosed with focal epilepsy (e.g. temporal or frontal lobe epilepsy) by a neurologist
- Maximum of three months since diagnosis
- Between and including the ages 16-65 years

Healthy controls

- No history of neurological or psychiatric illness or disease
- Between and including the ages 16-65 years
- No use of drugs or over four units of alcohol consumed in the preceding 48 hours

Exclusion Criteria

Patients with epilepsy

- Non-epileptic seizures
- Single seizures
- Primary generalised seizures
- Provoked seizures only (e.g. alcohol)

For peer review only - http://bmjopen.bmj.com/site/about/guidelines.xhtml

- Known inflammatory neurological condition (specifically multiple sclerosis or sarcoidosis)
- Acute symptomatic seizures (e.g. acute brain haemorrhage or brain injury)
- Progressive neurological disease (e.g. known brain tumour)
- Previous neurosurgery
- Concomitant infection
- Any other significant morbidity (physicians discretion)

Healthy controls

- Any neurological disease or illness
- Drug use or five or more units of alcohol consumed in the preceding 48 hours

MRI Criteria

All participants will be examined by a radiographer and will complete a safety checklist that is designed to identify whether a participant has internal bodily metal, which could pose a hazard during MRI scanning. All removable bodily metal will be removed before scanning. Standard MRI exclusion criteria include:

- Internal bodily metal, including
- Cardiac pacemaker or defibrillator
- Cochlear, otologic, or ear implant
- Any implant held in place by a magnet
- Implanted catheter, clamp, clips, valves, or other metal
- Presence or history of claustrophobia
- Pregnancy
- Unremovable bodily piercings or other metal

Sample size calculation

Taking into account that roughly 2/3 of patients with NDE are expected to achieve 12 months of remission within two years [21,22] approximately 72 patients with NDE (48/24 patients who achieve/do not achieve remission) and 48 controls are required to detect large effect sizes of 1.2 or above (large effects sizes are supported by our previous findings)[23], with power 90% and significance level of 0.001. Given the nature of the study and that a panel of biomarkers will be tested, a low significance level has been chosen to control for the false discovery rate (type I error) [24]. In the calculations we have also accounted for the ratio 2:1 for patients with NDE who achieve/do not achieve remission. After taking into account that ~25% patients will present with brain lesions,[25,26] and considering a potential attrition rate of 10%, a total of 107 patients with a new diagnosis of focal epilepsy will be recruited. Our experience leading multicentre clinical trials in patients with NDE,[27-29] and considering the inclusion criteria, is that it will take 30 months to recruit this number of patients from the WCFT and SRFT. The proposed sample size will also provide enough power to detect large effect sizes between NDE patients and controls with respect to neuropsychological performance [30] and therefore make it possible to address Objective 1.

Recruitment process

A summary of the recruitment process is shown in Figure 1. We will recruit participants attending WCFT and SRFT epilepsy clinics according to the aforementioned inclusion criteria. A clinical member of the research team (i.e. consultant neurologist, epilepsy nurse) will enquire whether eligible patients would be interested in participating in this study at the time of consultation in outpatient clinics. If so, the patient will be provided with an information sheet and consent form and allowed at least 48 hours to consider participation. The patient will then be contacted by telephone by a member of the research team (RT) to discuss

For peer review only - http://bmjopen.bmj.com/site/about/guidelines.xhtml

participation, and if the patient would still like to participate, an appointment will be made for the investigations. Patients will bring their signed and dated consent forms with them to their appointment. A member of the RT will confirm consent for each patient.

Healthy controls will be recruited from an existing volunteer register and advertisements placed on UoL notice boards. The recruitment of controls will be age-, sex- and educationally-matched. If we struggle to recruit educationally-matched controls, we will advertise to the local (Liverpool) community using online classified advertisements and community websites. A member of the RT will determine eligibility and interest of potential controls. Volunteers will be provided with a study information sheet and consent form via email. Eligible volunteers will be given an appointment for investigation. Control volunteers will bring their signed and dated consent forms with them to their appointment. A member of the RT will confirm consent for each control volunteer.

All participants will receive reimbursement of £100 for their participation in this study.

[Figure 1 about here.]

Participant withdrawal

Participants may withdraw their participation in this study at any time by contacting the RT. If participants withdraw from the study, information that has already been obtained will be kept in minimum personally-identifiable format to ensure that their privacy rights are safeguarded.

Outcomes

The primary treatment outcome variable is seizure outcome two years after diagnosis, which is a reliable time point and frequently used marker of pharmacoresistance.[31,32] Seizure freedom will be defined as a period of no seizures within the preceding 12 months at 2-year outcome, which aligns with current UK driving legislation.[33] The number and type of seizures experienced since the last follow up and current medication will be recorded by telephone by an epilepsy specialist nurse using a brief questionnaire adapted from the SANAD II clinical trial. In order to address Objective 1, we require control imaging and cognitive data from healthy participants, which will be compared with corresponding patient data. Based on previous findings,[25,26] ~25% of patients with NDE recruited are expected to have an identifiable lesion. Although the primary focus of this study will be on patients with MRI-negative NDE, as these represent the large majority of cases, having imaging and neuropsychological data from patients with lesional NDE will allow us to investigate whether the contribution of aberrant brain architecture and function is more significant than gross brain lesions for the prediction of cognitive dysfunction and treatment outcome. In brief, outcomes will consist of statistically significant differences in structural and functional brain connectivity and cognition between patients and controls as well as between patients with and without seizures two years after diagnosis.

Study phases

The study will last five years – from 1 October 2019 to 1 October 2024 – and be split into four phases. Five years are necessary given recruitment and follow-up objectives (Objective 2): we require a recruitment period long enough to recruit a sufficient number of patients with NDE and a follow up period long enough to establish likely seizure remission/pharmacoresistance. Figure 2 graphically illustrates the organisation of study phases.

[Figure 2 about here.]

Phase 1

[Ph1; month 1-3] is an initial 3-month period dedicated to project set-up, optimisation of the MRI protocol and psychologist training for proficient administration of the neuropsychology battery. MRI optimisation will include technical development MRI scanning of phantoms and human volunteers to ensure the MRI sequences are adequate for the study.

Phase 2

[Ph2; month 4-33] is a 30-month period that includes participant recruitment, and MRI, EEG, neuropsychological and blood serum and saliva data acquisition for all recruited participants. The imaging data acquired for all patients and controls will be processed

For peer review only - http://bmjopen.bmj.com/site/about/guidelines.xhtml

using image analysis techniques throughout Ph2. The final three months of Ph2 will be dedicated to the analysis of imaging markers of cognitive dysfunction to address Objective 1 once all imaging and neuropsychological data is collected (Ph2b).

Phase 3

[Ph3; month 10-57] is a 48-month patient follow-up period during which time all seizure outcome information will be recorded by telephone by a research nurse at the WCFT. Standardised assessment of patient seizure outcomes will be performed at 6, 12, 18 and 24 months after enrolment into the study. Although the 24-month assessment is the primary outcome time point for this study, we will endeavour to monitor patient status beyond the life of the grant award.

Phase 4

[Ph4; month 55-60] is the final 6-month period dedicated to addressing Objective 2 and Objective 3 when all outcome data is available using multivariate statistics and prognostic modelling.

Data acquisition

In total, we will perform 155 MRI, EEG, neuropsychological and blood/saliva sample investigations. Neuropsychological, MRI and blood/saliva sample data collection will be performed at LiMRC (Liverpool Magnetic Resonance Imaging Centre, Research Technology Building, UoL). The MRI protocol will include clinical sequences for diagnostic appraisal (see below), and a consultant neuroradiologist will review the scans of each participant as per standard clinical protocol. EEG data collection will take place at the WCFT and SRFT. Patients identified at SRFT will be transported from Manchester to Liverpool. A summary of the procedures for each participant is shown of Table 1.

LiMRC

Consent. Informed consent will be taken before assessments.

Neuropsychology. We will use a computerised neuropsychological battery (lasting up to two hours, including comfort breaks) that we have shown to be sensitive to cognitive deficits in people with NDE.[30,34] These will include components from the Wechsler Memory Scale Fourth Edition (WMS-IV),[35] Wechsler Adult Intelligence Scale Fourth Edition (WAIS-IV),[34,35] Delis-Kaplan Executive Function System (D-KEFS),[36,37] Patient Health Questionnaire 9 (PHQ-9),[38] Generalised Anxiety Disorder 7 (GAD-7),[39] The A-B Neuropsychological Assessment Schedule (ABNAS),[40] and Quality Of Life In Epilepsy (QOLIE-31) scale.[41] More specifically these assessment tools will be used to evaluate:

1. Auditory memory through story recall and recall of verbal pairs (WMS-IV)
2. Visual memory through reproduction of drawings and recall of designs (WMS-IV)
3. Working memory and attention through digit span and arithmetic tasks (WAIS-IV)
4. Processing speed through a coding and symbol search task (WAIS-IV)
5. Psychomotor speed through a finger tapping and visual reaction time task
6. Executive functioning through verbal fluency and colour-word interference tasks (D-KEFS)
7. Mood including depression (PHQ-9) and anxiety (GAD-7)
8. Perceived cognitive impairment (ABNAS)
9. Quality of life (QOLIE-31)

MRI scanning. The MRI protocol will be performed on a 3 T Siemens Prisma MRI at LiMRIC and will consist of the following sequences:

1. Conventional 2D T2-weighted fast spin echo and fast Fluid Attenuated Inversion Recovery scans, for incidental findings screening, and detection of gross pathology (together with localizer 11:00 minutes)
2. 3D T1-weighted MPRAGE scan with isotropic voxel size of 1 mm x 1 mm x 1mm (7:30 minutes)

For peer review only - http://bmjopen.bmj.com/site/about/guidelines.xhtml

3. fMRI verbal memory task scan adapted from Sidhu et al.[42]: whole brain echo planar imaging (EPI) sequence, with voxel size of 2 mm x 2 mm x 2mm, TR = 2.75; 10 concrete nouns shown for 3 seconds in 10 blocks of 30 seconds followed by a 15 second baseline period (fixation cross); participants indicate whether each word is pleasant or unpleasant (8:23 minutes)
4. Resting-state fMRI with eyes open with relaxed fixation on projected crosshair, whole brain echo planar imaging (EPI) sequence, with voxel size of 2 mm x 2 mm x 2mm, TR=2.5 (8:02 minutes)
5. Diffusion kurtosis imaging (DKI) sequence with 60 isotropically distributed gradient directions, three b values (b=0, 1000 and 2000) and maximum voxel size of 2 mm x 2 mm x 2mm (8:06 minutes)

Post scanning task. A verbal recognition task of words presented in the fMRI verbal memory task will be completed outside the scanner (<7 minutes).

Blood extraction. Blood will be collected for analysis in a Lithium- Heparin bottles or serum separator tubes (9mls). A maximum of 72 milliliters of blood (3 x 9ml vials) will be obtained from each participant. Samples will be obtained by a healthcare professional trained in phlebotomy. A standard operating procedure for blood sampling including aseptic technique will be utilised.

Saliva extraction. Samples of unstimulated saliva will be collected by soaking a sponge swab in the mouth of each participant until the swab is saturated with saliva. The swab will be inserted into a collection tube.

WCFT and SRFT

EEG. All participants will undergo a conventional clinical EEG, using 19 channels in 10-20 arrangement. Patients will be scanned in context of standard care in their respective trust (WCFT or SRFT) while controls will be scanned at the WCFT. Participant visiting time will last approximately 1 hour.

Data analysis

All MRI and EEG analysis techniques are automated and not subject to investigator bias.

MRI analysis

The MRI analysis procedures that will be carried out include (but will not be exclusive to):

Thalamocortical analysis. Our preliminary (unpublished) data has indicated that patients with NDE have structural changes in the thalamus. We will use DKI approaches to examine thalamic and thalamocortical connectivity. Mean DKI values will be obtained from spatially co-registered regions-of-interest (principally thalamocortical regions) in standard space. We will also apply diffusion[43] and resting-state functional MRI[44] independent component analysis techniques using FSL's MELODIC toolbox[45] (http://fsl.fmrib.ox.ac.uk/fsl/fslwiki/MELODIC) and in-house MATLAB scripts to identify abnormal structural and functional thalamocortical connectivity in patients relative to controls. We will also compare patient neuropsychological and treatment outcome groups using these approaches.

White matter tracts. Our recent publications have indicated that analysis of white matter tract diffusion has significance for predicting postsurgical seizure outcome in patients with chronic focal epilepsy[12,16] and that DKI is more sensitive to tract pathology than diffusion tensor imaging in epilepsy.[46] As white matter tracts constitute the structural connections within brain networks, we will determine DKI properties along the length of multi-lobar white matter tract bundles, using our recently reported methods.[46,47]

Large scale functional networks. Using our recently described resting-state analysis techniques,[23] we will identify and analyse features of the major resting-state networks, including the fronto-parietal attentional network, default mode network, salience network, and language network. All analyses will be performed using the Functional Connectivity Toolbox.[48]

Graph theory (Connectome). The development of whole brain connectomes [49] from diffusion MRI data have led to successful data-driven approaches to predict surgical responsiveness in patients with refractory focal epilepsy from members of our

For peer review only - http://bmjopen.bmj.com/site/about/guidelines.xhtml

group.[11,13-15] Connectome approaches also support the association between postoperative seizure control and thalamocortical connectivity.[14] Similar methods have been applied to resting-state functional MRI data to model functional connectome alterations in chronic focal epilepsy.[50] As per our recent connectomic studies, whole brain structural connectomes will be generated for each participant using T1-weighted and DKI data. T1-weighted data will be parcellated into multiple regions of interest (ROI; or nodes) using Freesurfer software (http://freesurfer.net). Structural connectivity between nodes will be determined using FSL's diffusion toolbox (http://fsl.fmrib.ox.ac.uk/fsl/fslwiki/FDT) for probabilistic fibre tracking applied to diffusion MRI. Structural connectomes will be generated using the Connectome Mapping Toolkit (http://www.connectome.ch). We will use graph theory to determine global and regional network configuration. Global network "small worldness" will be assessed, representing the ratio between average nodal clustering coefficients and as network efficiency. Regional clustering coefficient, efficiency and centrality will also be calculated for key brain areas associated with seizure onset and propagation, such as thalamocortical and limbic networks. We will generate resting-state functional connectomes using a similar approach to structural connectomes. Whereas for structural connectomes edges are represented by diffusion streamlines and kurtosis diffusion scalar metrics, functional connectomes will use fMRI time series correlations between each anatomical ROI to generate a temporal correlation matrix.

Other network approaches. Using DKI, T1, and resting-state fMRI data, we will apply network-based statistics to explore network alterations common to subgroups of patients,[51] and machine/deep learning algorithms to classify individual patient outcomes.[52]

Verbal memory fMRI. Following previous work,[42] we will explore the relationship between memory deficits and neural processing of verbal memory using task-based fMRI. An event-related analysis (FSL-FEAT) will be used to examine the neural correlates of successful subsequent memory formation, comparing memory encoding networks between patients and controls, as well as between seizure-free and drug-resistant epilepsy patients. Between-group ROI comparisons in temporal and extra-temporal regions will also be made with neuropsychological variables included as covariates into a General Linear Model. We hypothesise that altered neural processing of verbal memory will be observed in patients with memory-related deficits and that network organisation differences centred around temporal regions will be observed in patients with drug-resistant epilepsy.

EEG analysis

Resting-state EEG activity will be identified by a trained clinical EEG technician. Nodes in EEG networks will be defined as electrodes, and a range of measures of interdependence between electrodes will be explored. We will apply computer models of network dynamics to resting-state EEG data.[10] In order to address Objective 1 and Objective 2, we will analyse EEG network dynamics by mirroring the approach we will take with MRI (thalamocortical and connectome analysis); firstly, we will focus on thalamocortical physiological alterations by source-localising activity within thalamic and cortical regions and determine connectivity between regions using dynamic causal modelling.[53] Secondly, we will reconstruct resting-state EEG connectomes consistent with the resting-state functional MRI approach and determine network-based physiological differences between groups. We will also explore the inter-relation between EEG and MRI determined connectivity by performing DKI tractography seeded from nodes identified as aberrantly connected using EEG to determine whether abnormal physiological connectivity is related to abnormal structural connectivity. This approach has been adopted in patients with refractory epilepsy who underwent stereoelectroencephalography.[54]

Blood and saliva sample analysis

In the laboratory, blood samples will be centrifuged within 15 minutes of collection or stored overnight at 4˚c for centrifuge the following day. 250 µl aliquots will then be transferred to appropriate tubes and stored at approximately -80 degrees Celsius prior to bioanalysis. Saliva samples will be collected into an Eppendorf tube by squeezing the saturated swab using a syringe. The sample will be stored at -80 C freezer until assay. Blood and saliva samples will be analysed for inflammatory markers, HMGB1and brain-specific markers including microRNA. Inflammatory marker and HMGB1 expression analysis will be undertaken by ELISA and HMGB1 quantification will be made by liquid chromatography and mass spectrometry. Blood/saliva samples will be stored in the Liverpool University Biobank (LUB) freezer room, which is housed in the Research Technology building with LiMRIC.

For peer review only - http://bmjopen.bmj.com/site/about/guidelines.xhtml

Statistical analysis

We will explore the discriminatory effect of imaging biomarkers when taking into account their correlation structure and develop predictive models. (i) Cognitive dysfunction. Multivariate discriminant techniques will be used to identify structural/physiological brain measures that significantly differ between controls, patients with NDE who are cognitively normal and patients with NDE who are cognitively impaired (where impairment is defined by patient performance lower than two standard deviations [55] than that of the group of controls on respective neuropsychological tasks). We will also investigate the relationship between patient cognitive performance and imaging measures of architecture and network connectivity using multivariate regression analyses. (ii) Treatment outcome. Multivariate data techniques will be used to determine imaging measures that significantly differ between controls, patients who achieve remission over 12 months within two years, and patients who do not achieve remission within the same time period. Embedded multivariate techniques, such as least absolute shrinkage and selection operator (LASSO) and the support vector machines approaches (e.g. with radial and polynomial basis function kernels) will be applied to identify the panel of biomarkers with optimal discriminatory ability. For this process we will also consider patient demographic and clinical data, and presence of brain lesion. The variable selection algorithms will take into account the correlation between the variables, as well as variance differences across groups. To minimize the effect of possible over-fitting, penalty terms will be embedded in the variable selection algorithm to take into account model complexity. We will develop prognostic models for the assessment of outcome at two years using multivariate discriminant analysis.[56] Cross-validation and bootstrap techniques will be applied when appropriate.

Table 1 Summary of procedures

Procedure	Location	Duration	Number of examinations
1. Consent	Quiet assessment room, UoL	10 minutes	1
2. Neuropsychological evaluation	Quiet assessment room, UoL	2 hours, including comfort breaks	1
3. MRI	LiMRIC, UoL	1 hour, including safety examination and set up	1
4. Blood/saliva extraction	UoL	5 minutes	1
5. EEG	Neurophysiology, WCFT & SRFT	1 hour, including set up	1
6. Telephone questionnaire	Home	5 minutes	4 (6, 12, 18 and 24 months after scans)

ETHICS AND DISSEMINATION

Ethical approval

This study is approved by the North West, Liverpool East Research Ethics Committee (19/NW/0384) through the Integrated Research Application System (Project ID 260623). HRA approval was provided on 22/08/2019. The project is sponsored by the UoL (UoL001449) and funded by a UK Medical Research Council (MRC) research grant (MR/S00355X/1). The PI will ensure that the study is conducted in full accordance with approved protocols and that agreed modifications are disseminated to all relevant parties.

Confidentiality

Procedures for handling, processing, storage and destruction of your data are compliant with the Data Protection Act 1998. All EEG and MRI data will be anonymised prior to being exported from the WCFT, SRFT and LiMRIC, respectively. Personal information will not be identifiable from the imaging data. Names will be replaced with study ID numbers (EPINET001, EPINET002 etc), which can be backtracked to participant details using a key located at LiMRIC and only accessible to the primary care team. Storage eppendorfs will be labelled with the unique identifier only with no other patient information.

For peer review only - http://bmjopen.bmj.com/site/about/guidelines.xhtml

Similarly, all electronic neuropsychological data will be associated with study ID numbers. All documents associated with the study will be stored securely and only accessible by research staff and authorised personnel. Participant recruitment data will be monitored through study adoption by the NIHR portfolio.

Informed consent

All participants will be provided with a research information pack describing the nature and goals of the research, and study consent form, which must be completed, signed and dated. We will not recruit participants who lack capacity to provide informed consent (e.g. those with intellectual disability or dementia). Study consent forms will be retained and filed in a locked cupboard in the office of the PI. Information packs and consent forms will be given to people with epilepsy by a research nurse immediately after diagnosis in outpatient clinics. Information packs will be sent to healthy control volunteers via email or post. All participants will be given consent forms to complete at their first scanning appointment bring consent forms with them to their appointment for MRI and EEG scanning. The RT will the take the participant through the information sheet and consent form, explaining any aspects of the study that the participant is unclear about. Patients and controls will have as long as they require to consider their decision to volunteer for the research or not. The investigators contact details will be provided in the information pack.

Potential benefits and risks

There are no direct benefits to the participant. However, participation may lead to improved understanding of the aetiology, development and prognosis of epilepsy in the future.

MRI is considered a safe technique and scanning environment. The MRI system produces a high magnetic field, and it is necessary for the participants to remove all ferrometallic objects from their person before entering the scanning room. As per routine protocol, clinical members of staff screen participants for their suitability for scanning and fully debrief them after scanning. The MRI scanner is a noisy and confined environment, which may cause the participant to feel slight discomfort and claustrophobia. During scanning the subject will be monitored from the MRI control room by clinical staff and their heart-rate continuously monitored. If participants feel discomfort, scanning can be discontinued by pressing a distress button that the participant will be given before entering the scanner.

As indicated in the study information pack, scans will be reviewed to make sure that there is no brain pathology. Any MRI incidental finding will be reported to a consultant neuroradiologist. If the MRI finding is deemed to warrant further investigation, the participant's GP may be contacted. The percentage of unanticipated clinically serious brain abnormalities in healthy people is extremely low.

Venipuncture poses minimal risk of bruising or bleeding. All efforts will be made to minimise the risk of infection, appropriate training in infection control will be undertaken by all health care professionals.

There are no potential risks of EEG recordings. Neuropsychological evaluation could potentially lead to participant fatigue, frustration, or emotional disturbance. To obviate this, we will provide each participant with sufficient resting time between each assessment.

Patient and public involvement

No patients were directly involved in the design of this study. However, all participants will be fully debriefed regarding the goals and design of the research and will have the option of receiving a letter with a brief summary of the results at the end of the study.

Dissemination

We aim to produce high-impact peer-reviewed publications of the results of the study and present findings at national and international conferences, with exclusive access to the final study dataset for a period of six years. We will target epilepsy (e.g. European Congress for Epileptology, International Epilepsy Congress, International League Against Epilepsy (ILAE) UK Chapter, American Epilepsy Society) and neuroimaging (e.g. The Organisation of Human Brain Mapping, International Society for Magnetic Resonance in Medicine) conferences. The investigators will be involved in preparing manuscripts drafts, abstracts, press releases among any other publications arising from the study and will acknowledge that the study was funded by the MRC.

For peer review only

For peer review only - http://bmjopen.bmj.com/site/about/guidelines.xhtml

For each publication, only members of the research team who made a significant intellectual contribution to each piece of work will be considered as an author. This is in line with journal protocol. All authors share responsibility for the contents of the submitted manuscript.

ACKNOWLEDGEMENTS

The authors are thankful to members of staff in the Institution of Translational Medicine (ITM), UoL for internal peer review of the original grant proposal and study costings for the MRC grant application. The authors acknowledge all investigators at the planned recruitment sites (WCFT and SRFT) and Dr Kumar Das (Department of Neuroradiology, WCFT), Dr Shubhabrata Biswas (Department of Neuroradiology, WCFT), Dr Surjit Lyons-Nandra (Department of Neurophysiology, WCFT), Mrs Carole Hewitt (Neuroscience Research Centre, WCFT) and Ms Nicola Leek (ITM, UoL) for their support with the study.

FOOTNOTES

Author contributions: CdB contributed to the design of the MRI procedures and prepared the protocol for publication. MGF contributed to the statistical elements of the study and sample size calculations. GB, PM and NL helped to design the neuropsychological protocol. AM and RM contributed to the overall design of the study, leading on the setup of data collection methods at recruitment sites. LB and MR contributed to the analysis plan. SK conceived the study and led the development of the protocol. All authors provided critical intellectual input to the manuscript and have approved the final version for publication.

Funding: This work is supported by the MRC grant number MR/S00355X/1 and sponsored by the UoL (4720).

Disclaimer: The trial sponsor and funders had no role in trial design; collection, management, analysis and interpretation of data; writing of the report; and the decision to submit the report for publication, nor will they have ultimate authority over any of these activities. The views and opinions expressed herein are those of the authors and do not necessarily reflect those of the UoL, MRC, and NHS.

Technical appendix: Data will be made open access after being anonymised in accordance with the MRC's open access policy.

Competing interests: None declared.

Word Count: 5263 words.

REFERENCES

1 Epilepsy E. Action. *Epilepsy facts and terminology*. https://www.epilepsy.org.uk/press/facts (accessed 10 June 2019)
2 Baker GA. The psychosocial burden of epilepsy. *Epilepsia*. 2002;43:26-30.
3 Baker GA. Depression and suicide in adolescents with epilepsy. *Neurology*. 2006;66(66 suppl 3):S5-12.
4 Surges R, Sander JW. Sudden unexpected death in epilepsy: mechanisms, prevalence, and prevention. *Current opinion in neurology*. 2012;25(2):201-7.
5 Meador KJ, French J, Loring DW, Pennell PB. Disparities in NIH funding for epilepsy research. *Neurology*. 2011;77(13):1305-7.
6 Pohlmann-Eden B, Crocker CE, Schmidt MH. A conceptual framework for the use of neuroimaging to study and predict pharmacoresistance in epilepsy. *Epilepsia*. 2013;54:75-9.
7 Richardson MP. Large scale brain models of epilepsy: dynamics meets connectomics. *J Neurol Neurosurg Psychiatry*. 2012;83(12):1238-48.
8 Bernhardt BC, Bonilha L, Gross DW. Network analysis for a network disorder: the emerging role of graph theory in the study of epilepsy. *Epilepsy & Behavior*. 2015;50:162-70.
9 Terry JR, Benjamin O, Richardson MP. Seizure generation: the role of nodes and networks. *Epilepsia*. 2012;53(9):e166
10 Schmidt H, Woldman W, Goodfellow M, Chowdhury FA, Koutroumanidis M, Jewell S, Richardson MP, Terry JR. A computational biomarker of idiopathic generalized epilepsy from resting state EEG. *Epilepsia*. 2016;57(10):e200-4.

For peer review only - http://bmjopen.bmj.com/site/about/guidelines.xhtml

11Hutchings F, Han CE, Keller SS, Weber B, Taylor PN, Kaiser M. Predicting surgery targets in temporal lobe epilepsy through structural connectome based simulations. *PLoS computational biology*. 2015;11(12):e1004642.

12Keller SS, Richardson MP, Schoene-Bake JC, O'muircheartaigh J, Elkommos S, Kreilkamp B, Goh YY, Marson AG, Elger C, Weber B. Thalamotemporal alteration and postoperative seizures in temporal lobe epilepsy. *Annals of neurology*. 2015;77(5):760-74.

13Munsell BC, Wee CY, Keller SS, Weber B, Elger C, da Silva LA, Nesland T, Styner M, Shen D, Bonilha L. Evaluation of machine learning algorithms for treatment outcome prediction in patients with epilepsy based on structural connectome data. *Neuroimage*. 2015;118:219-30.

14Bonilha L, Helpern JA, Sainju R, Nesland T, Edwards JC, Glazier SS, Tabesh A. Presurgical connectome and postsurgical seizure control in temporal lobe epilepsy. *Neurology*. 2013;81(19):1704-10.

15Bonilha L, Jensen JH, Baker N, Breedlove J, Nesland T, Lin JJ, Drane DL, Saindane AM, Binder JR, Kuzniecky RI. The brain connectome as a personalized biomarker of seizure outcomes after temporal lobectomy. *Neurology*. 2015;84(18):1846-53.

16Keller SS, Glenn GR, Weber B, Kreilkamp BA, Jensen JH, Helpern JA, Wagner J, Barker GJ, Richardson MP, Bonilha L. Preoperative automated fibre quantification predicts postoperative seizure outcome in temporal lobe epilepsy. *Brain*. 2016;140(1):68-82.

17Maroso M, Balosso S, Ravizza T, et al. Toll-like receptor 4 and high-mobility group box-1 are involved in ictogenesis and can be targeted to reduce seizures. *Nature medicine*. 2010;14(4):413.

18Balosso S. Disulfide-containing high mobility group box-1 promotes N-methyl-D-aspartate receptor function and excitotoxicity by activating Toll-like receptor 4-dependent signaling in hippocampal neurons. Antioxid Redox Signal; 2014.

19Kelley MS, Jacobs MP, Lowenstein DH, NINDS Epilepsy Benchmark Stewards. The NINDS epilepsy research benchmarks. *Epilepsia*. 2009;50(3):579-82.

20Long C, Fureman B, Dingledine R. 2014 Epilepsy benchmarks: progress and opportunities. *Epilepsy currents*. 2016;16(3):179-81.

21Brodie, M.J., Road to refractory epilepsy: the Glasgow story. *Epilepsia*, 2013;54 Suppl 2:5-8.

22Mohanraj, R. and M.J. Brodie, Early predictors of outcome in newly diagnosed epilepsy. *Seizure*, 2013;22(5):333-44.

23Alonazi BK, Keller SS, Fallon N, Adams V, Das K, Marson AG, Sluming V. Resting-state functional brain networks in adults with a new diagnosis of focal epilepsy. *Brain and behavior*. 2019;9(1):e01168.

24Machin DC, Tan SB, Tan SH. *Sample size Tables for Clinical studies.* Wiley-Blackwell; 2009.

25Van Paesschen W, Duncan JS, Stevens JM, Connelly A. Etiology and early prognosis of newly diagnosed partial seizures in adults: a quantitative hippocampal MRI study. *Neurology*. 1997;49(3):753-7.

26Van Paesschen W, Duncan JS, Stevens JM, Connelly A. Longitudinal quantitative hippocampal magnetic resonance imaging study of adults with newly diagnosed partial seizures: one-year follow-up results. *Epilepsia*. 1998;39(6):633-9.

27Jacoby A, Lane S, Marson A, Baker GA, MESS Study Group. Relationship of clinical and quality of life trajectories following the onset of seizures: findings from the UK MESS Study. *Epilepsia*. 2011;52(5):965-74.

28Marson AG, Al-Kharusi AM, Alwaidh M, Appleton R, Baker GA, Chadwick DW, Cramp C, Cockerell OC, Cooper PN, Doughty J, Eaton B. The SANAD study of effectiveness of valproate, lamotrigine, or topiramate for generalised and unclassifiable epilepsy: an unblinded randomised controlled trial. *The Lancet*. 2007;369(9566):1016-26.

29SANADII [Internet]. Standard and New Antiepileptic Drugs Trial II Website [cited 2019 Jul 17]. Available from: http://www.sanad2.org.uk

30Taylor J, Kolamunnage-Dona R, Marson AG, Smith PE, Aldenkamp AP, Baker GA, SANAD Study Group. Patients with epilepsy: cognitively compromised before the start of antiepileptic drug treatment?. *Epilepsia*. 2010;51(1):48-56.

31Eriksson SH, Thom M, Symms MR, Focke NK, Martinian L, Sisodiya SM, Duncan JS. Cortical neuronal loss and hippocampal sclerosis are not detected by voxel-based morphometry in individual epilepsy surgery patients. *Human brain mapping*. 2009;30(10):3351-60.

32Mohanraj R, Brodie MJ. Early predictors of outcome in newly diagnosed epilepsy. *Seizure*. 2013;22(5):333-44.

For peer review only - http://bmjopen.bmj.com/site/about/guidelines.xhtml

33 Bonnett LJ, Powell GA, Smith CT, Marson AG. Risk of a seizure recurrence after a breakthrough seizure and the implications for driving: further analysis of the standard versus new antiepileptic drugs (SANAD) randomised controlled trial. *BMJ open*. 2017;7(7):e015868.

34 Baker GA, Taylor J, Aldenkamp AP. Newly diagnosed epilepsy: cognitive outcome after 12 months. *Epilepsia*. 2011;52(6):1084-91.

35 Drozdick LW, Holdnack JA, Weiss LG, Zhou X. Overview of the WAIS–IV/WMS–IV/ACS. *InWAIS-IV, WMS-IV, and ACS.* 2013; (pp. 1-73). Academic Press.

36 Swanson J. The Delis-Kaplan executive function system: a review. *Canadian Journal of School Psychology*. 2005;20(1-2):117-28.

37 Scarpina F, Tagini S. *The stroop color and word test. Frontiers in psychology*. 2017;8:557.

38 Fiest KM, Patten SB, Wiebe S, Bulloch AG, Maxwell CJ, Jetté N. Validating screening tools for depression in epilepsy. *Epilepsia*. 2014;55(10):1642-50.

39 Seo JG, Cho YW, Lee SJ, Lee JJ, Kim JE, Moon HJ, Park SP. Validation of the generalized anxiety disorder-7 in people with epilepsy: a MEPSY study. *Epilepsy & Behavior*. 2014;35:59-63.

40 Brooks J, Baker GA, Aldenkamp AP. The A-B Neuropsychological Assessment Schedule (ABNAS): the further refinement of a patient-based scale of patient-perceived cognitive functioning. *Epilepsy Research.* 2001;43:227-237.

41 Ridsdale L, Wojewodka G, Robinson E, Landau S, Noble A, Taylor S, Richardson M, Baker G, Goldstein LH, SMILE Team. Characteristics associated with quality of life among people with drug-resistant epilepsy. Journal of neurology. 2017;264(6):1174-84.93

42 Sidhu MK, Stretton J, Winston GP, Bonelli S, Centeno M, Vollmar C, Symms M, Thompson PJ, Koepp MJ, Duncan JS. A functional magnetic resonance imaging study mapping the episodic memory encoding network in temporal lobe epilepsy. *Brain*. 2013;136(6):1868-88.

43 O'Muircheartaigh J, Vollmar C, Traynor C, Barker GJ, Kumari V, Symms MR, Thompson P, Duncan JS, Koepp MJ, Richardson MP. Clustering probabilistic tractograms using independent component analysis applied to the thalamus. *Neuroimage*. 2011;54(3):2020-32

44 O'Muircheartaigh J, Keller SS, Barker GJ, Richardson MP. White matter connectivity of the thalamus delineates the functional architecture of competing thalamocortical systems. *Cerebral Cortex*. 2015;25(11):4477-89.

45 Beckmann CF, Smith SM. Tensorial extensions of independent component analysis for multisubject FMRI analysis. *Neuroimage*. 2005;25(1):294-311.

46 Glenn GR, Jensen JH, Helpern JA, Spampinato MV, Kuzniecky R, Keller SS, Bonilha L. Epilepsy-related cytoarchitectonic abnormalities along white matter pathways. *J Neurol Neurosurg Psychiatry*. 2016;87(9):930-6.

47 Kreilkamp BA, Weber B, Richardson MP, Keller SS. Automated tractography in patients with temporal lobe epilepsy using TRActs Constrained by UnderLying Anatomy (TRACULA). *NeuroImage*: Clinical. 2017;14:67-76.

48 Whitfield-Gabrieli S, Nieto-Castanon A. Conn: a functional connectivity toolbox for correlated and anticorrelated brain networks. *Brain connectivity*. 2012;2(3):125-41.

49 Sporns O. *Discovering the human connectome*. MIT press; 2012.

50 Liao W, Ji GJ, Xu Q, Wei W, Wang J, Wang Z, Yang F, Sun K, Jiao Q, Richardson MP, Zang YF. Functional connectome before and following temporal lobectomy in mesial temporal lobe epilepsy. *Scientific reports*. 2016;6:23153.

51 Zalesky A, Fornito A, Bullmore ET. Network-based statistic: identifying differences in brain networks. *Neuroimage*. 2010;53(4):1197-207.

52 Munsell BC, Wee CY, Keller SS, Weber B, Elger C, da Silva LA, Nesland T, Styner M, Shen D, Bonilha L. Evaluation of machine learning algorithms for treatment outcome prediction in patients with epilepsy based on structural connectome data. *Neuroimage*. 2015;118:219-30.

53 Kiebel SJ, Garrido MI, Moran RJ, Friston KJ. Dynamic causal modelling for EEG and MEG. *Cognitive neurodynamics*. 2008;2(2):121.

54 Besson P, Bandt SK, Proix T, Lagarde S, Jirsa VK, Ranjeva JP, Bartolomei F, Guye M. Anatomic consistencies across epilepsies: a stereotactic-EEG informed high-resolution structural connectivity study. *Brain*. 2017;140(10):2639-52.

For peer review only - http://bmjopen.bmj.com/site/about/guidelines.xhtml

55Goldman JG, Holden S, Bernard B, Ouyang B, Goetz CG, Stebbins GT. Defining optimal cutoff scores for cognitive impairment using Movement Disorder Society Task Force criteria for mild cognitive impairment in Parkinson's disease. *Movement Disorders*. 2013;28(14):1972-9.

56Hughes DM, Komárek A, Bonnett LJ, Czanner G, García-Fiñana M. Dynamic classification using credible intervals in longitudinal discriminant analysis. *Statistics in medicine*. 2017;36(24):3858-74.

LIST OF FIGURES

1 The recruitment process 16
2 Organisation of the study phases 17

For peer review only

For peer review only - http://bmjopen.bmj.com/site/about/guidelines.xhtml

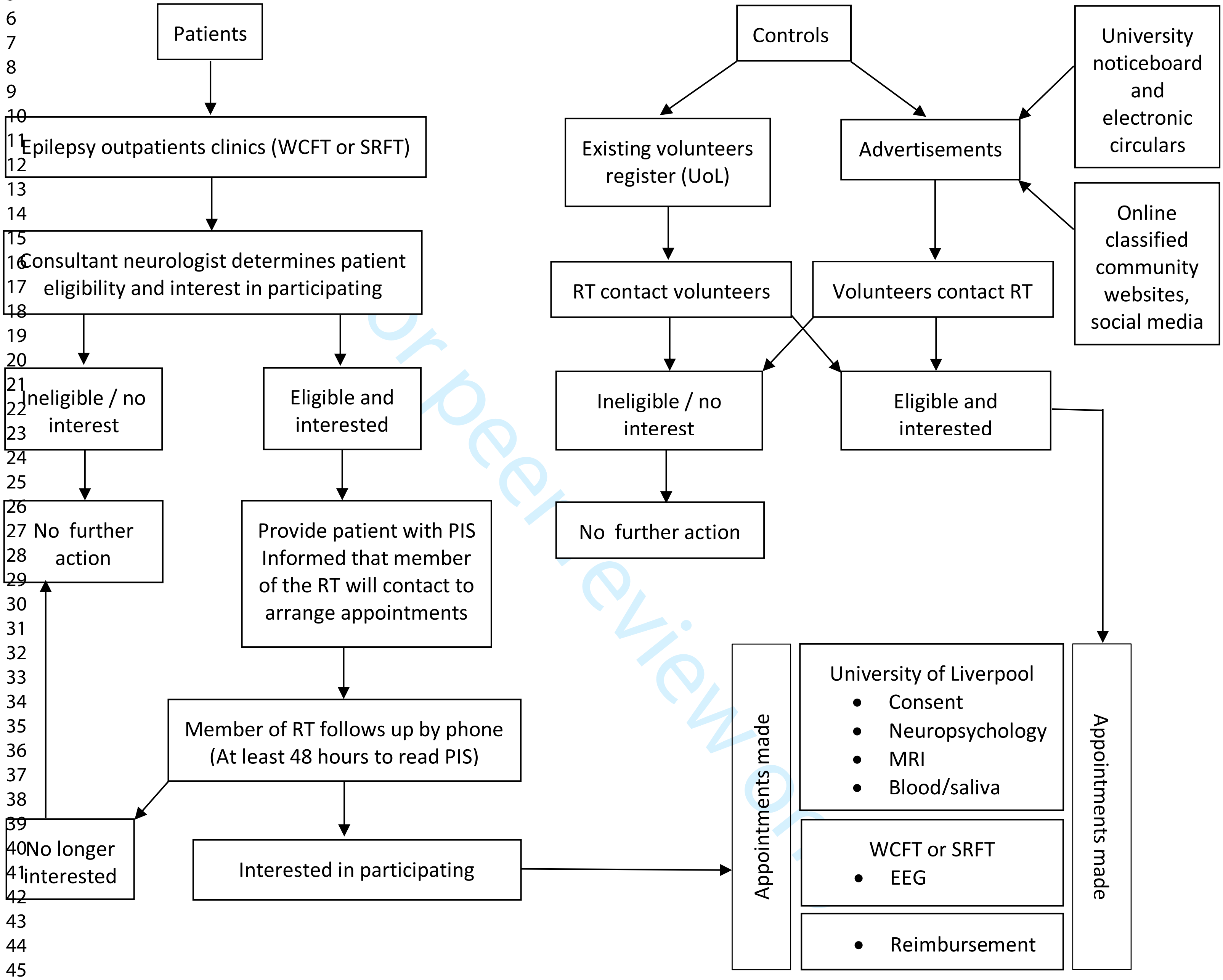
Patients
Epilepsy outpatients clinics (WCFT or SRFT)
Consultant neurologist determines patient eligibility and interest in participating
Ineligible / no interest
Eligible and interested
No further action
Provide patient with PIS Informed that member of the RT will contact to arrange appointments
Member of RT follows up by phone (At least 48 hours to read PIS)
No longer interested
Interested in participating
Controls
Existing volunteers register (UoL)
Advertisements
University noticeboard and electronic circulars
Online classified community websites, social media
RT contact volunteers
Volunteers contact RT
Ineligible / no interest
Eligible and interested
No further action
Appointments made
University of Liverpool
Consent
Neuropsychology
MRI
Blood/saliva
WCFT or SRFT
EEG
Reimbursement
Appointments made

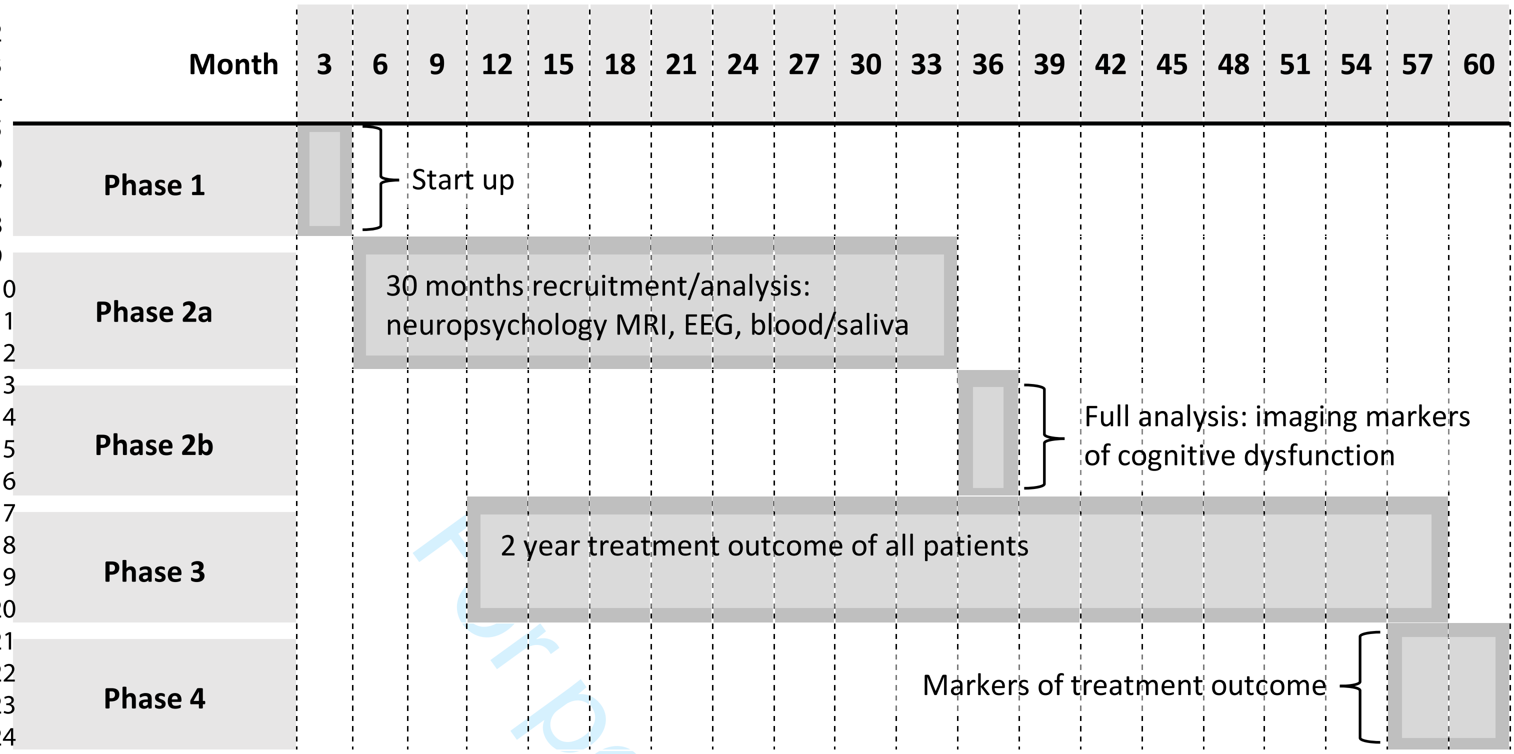

For peer review only

Reporting checklist for protocol of a clinical trial.

Based on the SPIRIT guidelines.

Instructions to authors

Complete this checklist by entering the page numbers from your manuscript where readers will find each of the items listed below.

Your article may not currently address all the items on the checklist. Please modify your text to include the missing information. If you are certain that an item does not apply, please write "n/a" and provide a short explanation.

Upload your completed checklist as an extra file when you submit to a journal.

In your methods section, say that you used the SPIRITreporting guidelines, and cite them as:

Chan A-W, Tetzlaff JM, Altman DG, Laupacis A, Gøtzsche PC, Krleža-Jerić K, Hróbjartsson A, Mann H, Dickersin K, Berlin J, Doré C, Parulekar W, Summerskill W, Groves T, Schulz K, Sox H, Rockhold FW, Rennie D, Moher D. SPIRIT 2013 Statement: Defining standard protocol items for clinical trials. Ann Intern Med. 2013;158(3):200-207

		Reporting Item	Page Number
**Administrative information**			
Title	#1	Descriptive title identifying the study design, population, interventions, and, if applicable, trial acronym	2
Trial registration	#2a	Trial identifier and registry name. If not yet registered, name of intended registry	2
Trial registration: data set	#2b	All items from the World Health Organization Trial Registration Data Set	n/a
Protocol version	#3	Date and version identifier	2
Funding	#4	Sources and types of financial, material, and other support	2
Roles and responsibilities: contributorship	#5a	Names, affiliations, and roles of protocol contributors	12

For peer review only

Roles and responsibilities: sponsor contact information	#5b	Name and contact information for the trial sponsor	2
Roles and responsibilities: sponsor and funder	#5c	Role of study sponsor and funders, if any, in study design; collection, management, analysis, and interpretation of data; writing of the report; and the decision to submit the report for publication, including whether they will have ultimate authority over any of these activities	12
Roles and responsibilities: committees	#5d	Composition, roles, and responsibilities of the coordinating centre, steering committee, endpoint adjudication committee, data management team, and other individuals or groups overseeing the trial, if applicable (see Item 21a for data monitoring committee)	12
**Introduction**			
Background and rationale	#6a	Description of research question and justification for undertaking the trial, including summary of relevant studies (published and unpublished) examining benefits and harms for each intervention	2-3
Background and rationale: choice of comparators	#6b	Explanation for choice of comparators	3
Objectives	#7	Specific objectives or hypotheses	3
Trial design	#8	Description of trial design including type of trial (eg, parallel group, crossover, factorial, single group), allocation ratio, and framework (eg, superiority, equivalence, non-inferiority, exploratory)	3
**Methods: Participants, interventions, and outcomes**			
Study setting	#9	Description of study settings (eg, community clinic, academic hospital) and list of countries where data will be	4

For peer review only

		collected. Reference to where list of study sites can be obtained	
Eligibility criteria	#10	Inclusion and exclusion criteria for participants. If applicable, eligibility criteria for study centres and individuals who will perform the interventions (eg, surgeons, psychotherapists)	4
Interventions: description	#11a	Interventions for each group with sufficient detail to allow replication, including how and when they will be administered	6-8
Interventions: modifications	#11b	Criteria for discontinuing or modifying allocated interventions for a given trial participant (eg, drug dose change in response to harms, participant request, or improving / worsening disease)	n/a Observational cohort study
Interventions: adherance	#11c	Strategies to improve adherence to intervention protocols, and any procedures for monitoring adherence (eg, drug tablet return; laboratory tests)	n/a Observational cohort study
Interventions: concomitant care	#11d	Relevant concomitant care and interventions that are permitted or prohibited during the trial	n/a<br><br>Observational cohort study
Outcomes	#12	Primary, secondary, and other outcomes, including the specific measurement variable (eg, systolic blood pressure), analysis metric (eg, change from baseline, final value, time to event), method of aggregation (eg, median, proportion), and time point for each outcome. Explanation of the clinical relevance of chosen efficacy and harm outcomes is strongly recommended	6
Participant timeline	#13	Time schedule of enrolment, interventions (including any run-ins and washouts), assessments, and visits for participants. A schematic diagram is highly recommended (see Figure)	6
Sample size	#14	Estimated number of participants needed to achieve study objectives and how it was determined, including clinical and statistical assumptions supporting any sample size calculations	5

For peer review only

Recruitment	#15	Strategies for achieving adequate participant enrolment to reach target sample size	5
**Methods: Assignment of interventions (for controlled trials)**			
Allocation: sequence generation	#16a	Method of generating the allocation sequence (eg, computer-generated random numbers), and list of any factors for stratification. To reduce predictability of a random sequence, details of any planned restriction (eg, blocking) should be provided in a separate document that is unavailable to those who enrol participants or assign interventions	n/a<br><br>Observational cohort study
Allocation concealment mechanism	#16b	Mechanism of implementing the allocation sequence (eg, central telephone; sequentially numbered, opaque, sealed envelopes), describing any steps to conceal the sequence until interventions are assigned	n/a<br><br>Observational cohort study
Allocation: implementation	#16c	Who will generate the allocation sequence, who will enrol participants, and who will assign participants to interventions	n/a<br><br>Observational cohort study
Blinding (masking)	#17a	Who will be blinded after assignment to interventions (eg, trial participants, care providers, outcome assessors, data analysts), and how	n/a<br><br>Observational cohort study
Blinding (masking): emergency unblinding	#17b	If blinded, circumstances under which unblinding is permissible, and procedure for revealing a participant's allocated intervention during the trial	n/a<br><br>Observational cohort study
**Methods: Data collection, management, and analysis**			
Data collection plan	#18a	Plans for assessment and collection of outcome, baseline, and other trial data, including any related processes to promote data quality (eg, duplicate measurements, training	5-8

For peer review only

		of assessors) and a description of study instruments (eg, questionnaires, laboratory tests) along with their reliability and validity, if known. Reference to where data collection forms can be found, if not in the protocol	
Data collection plan: retention	#18b	Plans to promote participant retention and complete follow-up, including list of any outcome data to be collected for participants who discontinue or deviate from intervention protocols	5
Data management	#19	Plans for data entry, coding, security, and storage, including any related processes to promote data quality (eg, double data entry; range checks for data values). Reference to where details of data management procedures can be found, if not in the protocol	10
Statistics: outcomes	#20a	Statistical methods for analysing primary and secondary outcomes. Reference to where other details of the statistical analysis plan can be found, if not in the protocol	8-10
Statistics: additional analyses	#20b	Methods for any additional analyses (eg, subgroup and adjusted analyses)	8-10
Statistics: analysis population and missing data	#20c	Definition of analysis population relating to protocol non-adherence (eg, as randomised analysis), and any statistical methods to handle missing data (eg, multiple imputation)	8-10
**Methods: Monitoring**			
Data monitoring: formal committee	#21a	Composition of data monitoring committee (DMC); summary of its role and reporting structure; statement of whether it is independent from the sponsor and competing interests; and reference to where further details about its charter can be found, if not in the protocol. Alternatively, an explanation of why a DMC is not needed	11
Data monitoring: interim analysis	#21b	Description of any interim analyses and stopping guidelines, including who will have access to these interim results and make the final decision to terminate the trial	n/a<br>No interim analysis planned

For peer review only

Harms	#22	Plans for collecting, assessing, reporting, and managing solicited and spontaneously reported adverse events and other unintended effects of trial interventions or trial conduct	11
Auditing	#23	Frequency and procedures for auditing trial conduct, if any, and whether the process will be independent from investigators and the sponsor	n/a<br><br>No auditing trial conduct
**Ethics and dissemination**			
Research ethics approval	#24	Plans for seeking research ethics committee / institutional review board (REC / IRB) approval	10
Protocol amendments	#25	Plans for communicating important protocol modifications (eg, changes to eligibility criteria, outcomes, analyses) to relevant parties (eg, investigators, REC / IRBs, trial participants, trial registries, journals, regulators)	10
Consent or assent	#26a	Who will obtain informed consent or assent from potential trial participants or authorised surrogates, and how (see Item 32)	10
Consent or assent: ancillary studies	#26b	Additional consent provisions for collection and use of participant data and biological specimens in ancillary studies, if applicable	n/a
Confidentiality	#27	How personal information about potential and enrolled participants will be collected, shared, and maintained in order to protect confidentiality before, during, and after the trial	10-11
Declaration of interests	#28	Financial and other competing interests for principal investigators for the overall trial and each study site	12
Data access	#29	Statement of who will have access to the final trial dataset, and disclosure of contractual agreements that limit such access for investigators	11
Ancillary and post trial care	#30	Provisions, if any, for ancillary and post-trial care, and for compensation to those who suffer harm from trial participation	n/a

For peer review only

			Observational cohort study
Dissemination policy: trial results	[#31a](#)	Plans for investigators and sponsor to communicate trial results to participants, healthcare professionals, the public, and other relevant groups (eg, via publication, reporting in results databases, or other data sharing arrangements), including any publication restrictions	11
Dissemination policy: authorship	[#31b](#)	Authorship eligibility guidelines and any intended use of professional writers	11
Dissemination policy: reproducible research	[#31c](#)	Plans, if any, for granting public access to the full protocol, participant-level dataset, and statistical code	n/a
**Appendices**			
Informed consent materials	[#32](#)	Model consent form and other related documentation given to participants and authorised surrogates	n/a
Biological specimens	[#33](#)	Plans for collection, laboratory evaluation, and storage of biological specimens for genetic or molecular analysis in the current trial and for future use in ancillary studies, if applicable	9

None The SPIRIT checklist is distributed under the terms of the Creative Commons Attribution License CC-BY-ND 3.0. This checklist can be completed online using https://www.goodreports.org/, a tool made by the EQUATOR Network in collaboration with Penelope.ai

For peer review only
