# Supplementary material for: Investigating imaging network markers of cognitive dysfunction and pharmacoresistance in newly diagnosed epilepsy: a protocol for an observational cohort study in the UK
Source: BMJ Open. 2019 Oct 16;9(10):e034347. doi: 10.1136/bmjopen-2019-034347 (PMC6797398; doi:10.1136/bmjopen-2019-034347)
Supplement: Reviewer comments [file bmjopen-2019-034347.reviewer_comments.pdf]

PEER REVIEW HISTORY

BMJ Open publishes all reviews undertaken for accepted manuscripts. Reviewers are asked to complete a checklist review form (**http://bmjopen.bmj.com/site/about/resources/checklist.pdf**) and are provided with free text boxes to elaborate on their assessment. These free text comments are reproduced below.

ARTICLE DETAILS

**TITLE (PROVISIONAL)**	A protocol for an observational cohort study in the UK investigating imaging network markers of cognitive dysfunction and pharmacoresistance in newly diagnosed epilepsy.
**AUTHORS**	de Bézenac, Christophe; Garcia-Finana, Marta; Baker, Gus; Moore, Perry; Leek, Nicola; Mohanraj, Rajiv; Bonilha, Leonardo; Richardson, Mark; Marson, Anthony; Keller, Simon

This article was not externally reviewed at BMJ Open. Protocols that have been independently assessed prior to submission to BMJ Open are usually fast-tracked to publication on the grounds that further substantial changes will not be possible. This independent assessment will usually be external, independent review for both a substantial grant award from a non-commercial or government funder and ethics approval.
